# COVID-19 prevention and treatment: A critical analysis of chloroquine and hydroxychloroquine clinical pharmacology

**DOI:** 10.1371/journal.pmed.1003252

**Published:** 2020-09-03

**Authors:** Nicholas J. White, James A. Watson, Richard M. Hoglund, Xin Hui S. Chan, Phaik Yeong Cheah, Joel Tarning

**Affiliations:** 1 Mahidol-Oxford Tropical Medicine Research Unit, Faculty of Tropical Medicine, Mahidol University, Bangkok, Thailand; 2 Centre for Tropical Medicine and Global Health, Nuffield Department of Medicine, University of Oxford, Oxford, United Kingdom; 3 Hospital for Tropical Diseases, University College London Hospitals NHS Foundation Trust, London, United Kingdom

## Abstract

Nicholas White and coauthors discuss chloroquine and hydroxychloroquine pharmacology in the context of possible treatment of SARS-CoV-2 infection.

**Summary points**

- Chloroquine and hydroxychloroquine have been used for over 60 years in the treatment of malaria, amoebic liver abscess, and several rheumatological conditions, but their clinical pharmacology is not well understood. COVID-19 is a new potential indication, although these drugs have only moderate in vitro activity against the SARS-CoV-2 virus and there is no convincing evidence at this time of significant clinical efficacy.
- Chloroquine and hydroxychloroquine both have unusual pharmacokinetic properties with enormous apparent volumes of distribution (chloroquine > hydroxychloroquine) and very slow elimination from the body (terminal elimination half-lives > 1 month).
- The free plasma concentrations that drive potentially serious adverse reactions (hypotension, cardiac conduction disturbances, delayed ventricular repolarization, and neurotoxicity) are determined largely by distribution processes.
- Hydroxychloroquine was slightly safer than chloroquine in preclinical testing and is considered better tolerated over the long term. Both drugs are dangerous when overdosed, and parenteral administration needs careful control.
- There are different salts, each with a different base equivalent. This has led to confusion and sometimes mistakes in dosing. As different salts are available in different places, malaria treatment is usually recommended in terms of base equivalent. Tablets of the two most widely available forms, chloroquine diphosphate 250 mg salt and hydroxychloroquine sulphate 200 mg salt, both contain 155 mg base.
- The pro-arrhythmic and anti-arrhythmic effects of chloroquine and hydroxychloroquine have been poorly characterised, although the majority of evidence for current regimens is very reassuring. Arrhythmia risks have been inferred from QT prolongation rather than observed.
- We used available pharmacokinetic information from healthy volunteers, the treatment of malaria, the chronic treatment of rheumatological conditions, and the toxicokinetics of chloroquine in self-poisoning to predict exposures and safety margins in the high-dose COVID-19 prevention and treatment regimens that have been evaluated.
- These regimens are predicted to have reasonable safety margins. Using lower doses risks failing to identify any putative benefit in this potentially lethal infection. Dose regimens should aim to avoid reaching whole-blood concentrations over 10 μM (3.2 μg/mL), which correspond approximately to plasma concentrations >3 μM (1 μg/mL).
- Large, well-conducted randomised clinical trials with appropriate monitoring are required to determine if chloroquine and hydroxychloroquine have preventive or treatment efficacy in COVID-19 and acceptable safety. Current recommendations for their use outside of clinical trials are not justified at this time.

## Introduction

**Fig 1:**
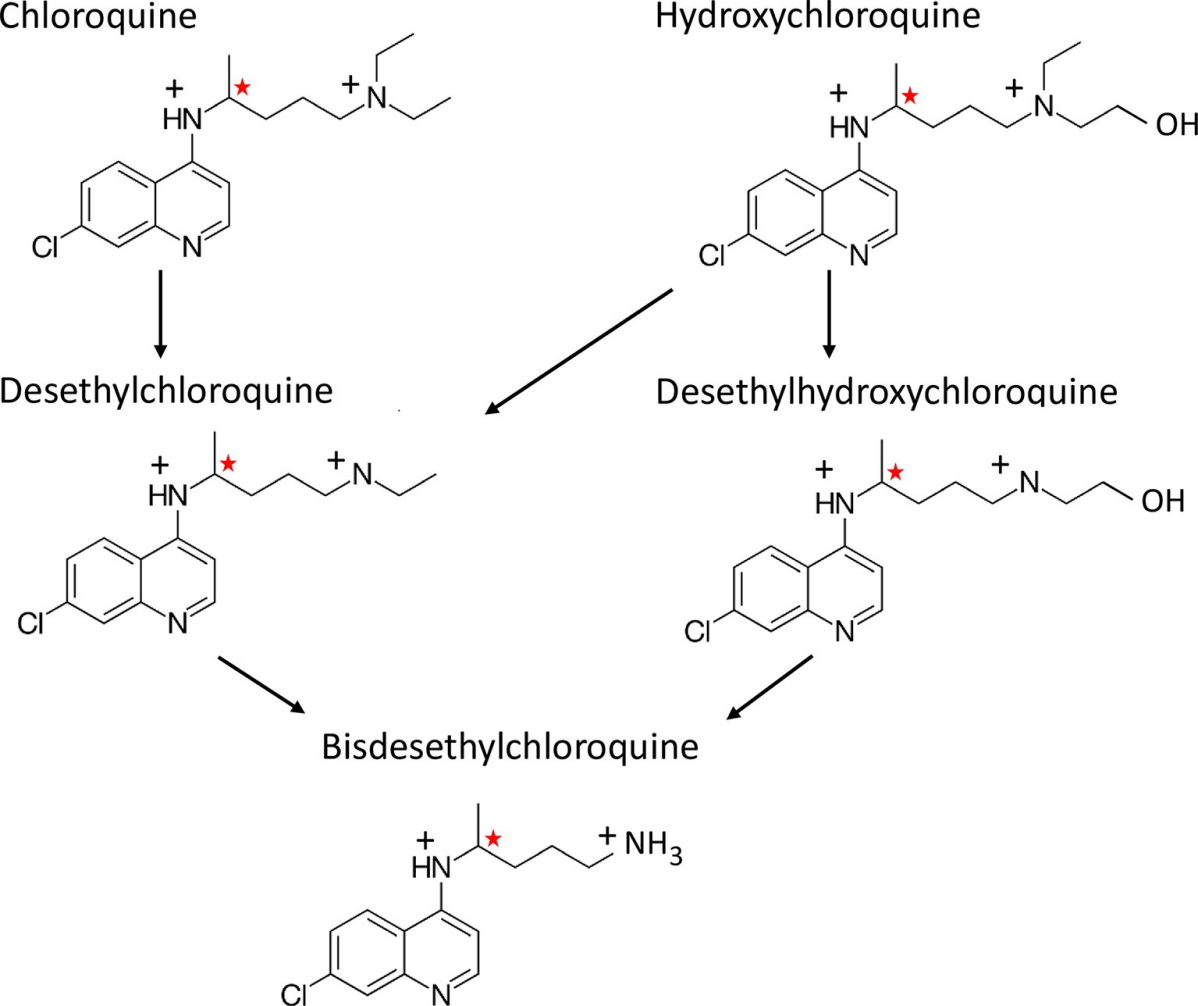
The metabolism of chloroquine and hydroxychloroquine. The red stars indicate chiral centres.

Chloroquine [7-chloro-4-[4-(diethylamino)-1-methylbutyl]amino] quinoline is a 4-aminoquinoline compound discovered in Germany in 1934 as part of a research programme to develop new antimalarial drugs [1, 2]. Hydroxychloroquine, in which one of the ethyl groups in the alkyl side chain is hydroxylated, was synthesized in 1946 (Fig 1). Early clinical pharmacology assessments in the United States of America characterised the safety, tolerability, and antimalarial efficacy of chloroquine. By the early 1950s, chloroquine had become the treatment of choice for all malaria throughout the world, and hundreds of metric tonnes (corresponding to nearly 100 million malaria treatment doses) were dispensed annually [3]. Industrial production peaked in 2004. In the last quarter of 2004, China alone reported production of over 400 tonnes [4]. Thus, well over 5 billion treatments have been dispensed worldwide. Chloroquine can claim to be among the drugs to which humans have been most exposed.

Today, although *Plasmodium falciparum* is resistant to chloroquine everywhere except in Haiti and Central America north of the Panama Canal, chloroquine remains a first-line treatment option for non-falciparum malaria [5]. Chloroquine was also used extensively in chemoprophylaxis to prevent malaria, including in pregnancy [5–7]. In addition, chloroquine proved to be effective in the treatment of amoebic liver abscesses and to have important anti-inflammatory properties. Hydroxychloroquine was developed more for its use in rheumatological conditions [8]. It is generally considered to be slightly safer than chloroquine (S1 Table shows animal data) [9], although the evidence to support this in humans is not strong. The treatment of malaria required a short-course regimen (usual total dose 25 mg base/kg, up to 50 mg/kg) over 2 or 3 days, whereas higher total doses (10 mg base/kg daily for 2 days followed by 5 mg base/kg daily for 2–3 weeks) were used for hepatic amoebiasis [10]. This review focuses on the clinical pharmacology of these two 4-aminoquinolines in relation to potential preventive and treatment use in COVID-19. It does not review in detail the evidence for antiviral activity or for immune modulation, which could also contribute to benefit in ameliorating the inflammatory manifestations of COVID-19.

## Clinical pharmacokinetics

Even in the 1940s, when drug measurement was in its infancy, it was clear that the 4-aminoquinolines had unusual pharmacokinetic properties [2]. Absorption after oral administration was rapid and generally reliable, but the total apparent volume of distribution was enormous (>100 L/kg) reflecting extensive tissue binding [11–29]. Hydroxychloroquine may have a smaller total apparent volume of distribution but otherwise similar absorption, distribution, and elimination kinetics [9, 24–27, 30–40]. Estimates for the terminal elimination half-life lengthened as the less sensitive spectrophotometric assays were replaced by high-performance liquid chromatography (HPLC) methods (i.e., with increasing sensitivity a greater proportion of the terminal phase could be characterised). The quoted average values for terminal phase elimination half-lives for chloroquine (approximately 38 days) and hydroxychloroquine (approximately 54 days) may not represent significant differences [13, 14, 41]. Thus, the initial plasma or whole-blood concentration profile in the treatment of acute illness is determined mainly by distribution processes and not by drug elimination [13, 16, 18, 20]. This is critical to understanding the relationship between dosing and concentration profiles and the associated risks with short-course treatments. In treatment regimens, the initial (loading) doses are designed to ‘fill’ the body so that concentrations that would take weeks to achieve without a loading dose are achieved as soon as safely possible.

**Fig 2:**
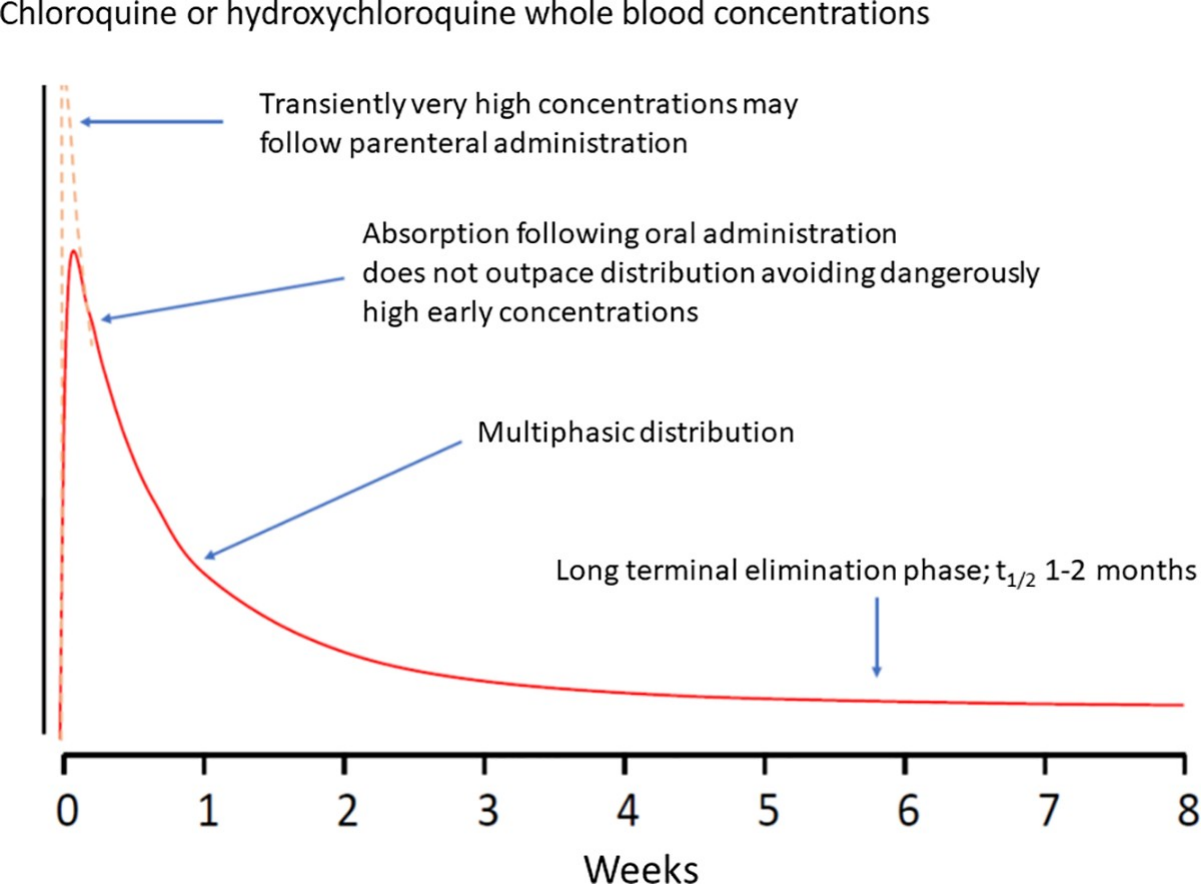
Example of a general whole-blood or plasma concentration-time profile for chloroquine or hydroxychloroquine.

The dosing and spacing of the loading dose aims to provide sufficient time for chloroquine or hydroxychloroquine to diffuse out from the relatively small central ‘compartment’ and thereby avoid accumulation to transient very high concentrations that are potentially toxic (Fig 2) [16]. Free chloroquine equilibrates at different rates with different tissues and cellular components, but the vascular smooth muscle and cardiac muscle seem to be in rapid equilibrium such that haemodynamic and cardiac electrophysiological changes occur almost synchronously with blood concentrations, so there is little hysteresis in the cardiovascular concentration-effect relationship [16].

Orally administered chloroquine is well absorbed, even in unconscious patients [12–14, 17, 20]. Chloroquine is absorbed very rapidly following subcutaneous or intramuscular injection such that absorption may outpace distribution and, with doses of 5 mg base/kg or more, transiently toxic concentrations may occur [15, 20]. To avoid this, intravenous chloroquine is administered by constant-rate infusion, and subcutaneous or intramuscular chloroquine is given in small (2.5–3.5 mg base/kg), frequent injections [18, 20]. The principal metabolite, desethylchloroquine, has approximately equivalent antimalarial and other biological activities [42–44]. Measurement of chloroquine and hydroxychloroquine in blood is complicated by extensive binding within leukocytes and platelets and, to a lesser extent, erythrocytes [45, 46]. As a result, plasma levels (with adequate centrifugation of blood samples at 2,000*g* for >10 minutes) are half those in serum. Reported values for the ratio of whole blood to plasma for both drugs range from 3 to 10 with substantial variability in the published literature; see S2 Table [47]. For these reasons, whole blood is the preferred matrix for pharmacokinetic studies. Early spectrophotometric assays used before the 1990s lacked specificity in distinguishing the desethylated metabolites and, although they were accurate at higher concentrations, they were relatively insensitive [48]. Most recent studies have used HPLC with UV or tandem mass spectrometry (MS/MS) detection.

Chloroquine has been formulated as sulphate, phosphate, and hydrochloride salts and is prescribed for malaria in weights of base content (S1 Text). Various liquid formulations are available for paediatric use. Chloroquine can be given parenterally, orally, or by suppository [5]. Hydroxychloroquine has been made in a parenteral formulation, but the usual form is a tablet of the sulphate salt.

## Elimination

Renal elimination accounts for 20%–55% of total clearance. Using spectrophotometric assays, McChesney and colleagues estimated that 55% of an oral chloroquine dose was eliminated in the urine with 70% as unchanged drug, 23% as desethylchloroquine, 1%–2% as bisdesethylchloroquine, and the remainder as other metabolites [12]. Hydroxychloroquine is also desethylated and dehydroxylated so that desethylchloroquine and bisdesethylchloroquine are formed as well as desethylhydroxychloroquine (Fig 1). N-desethylation is mediated by CYP 2D6, 3A4, 3A5, and 2C8 isoforms [49]. CYP2D6 polymorphisms do affect the steady-state ratio of parent drug to active metabolite [39], but this is of uncertain clinical relevance. Clinically significant pharmacokinetic drug-drug interactions with hydroxychloroquine and chloroquine have not been reported. Accumulation occurs slowly with repeated dosing (as in rheumatological conditions or continuous antimalarial or COVID-19 chemoprophylaxis) because of the very slow terminal elimination rate (e.g., S1 Fig). Higher levels are reached in patients with renal failure [37, 40].

## Antiviral and antimalarial activities

The 4-aminoquinolines inhibit the pH-dependent steps of replication of a broad range of viruses (including flaviviruses, retroviruses, and coronaviruses) [29, 50–54]. The exact mechanism of antiviral action is unclear, as these drugs may interfere with nearly every step of cellular infection and replication—i.e., viral fusion, viral penetration, nucleic acid replication, viral protein glycosylation, virus assembly, new virus particle transport, and virus release [29, 50, 51]. The 4-aminoquinolines may inhibit binding of the SARS-CoV-2 spike protein to gangliosides and sialic acid residues around the ACE-2 receptor [55]. Activities (EC_50_s) against the SARS-CoV-2 virus are in the low micromolar range, which represents the upper end of the safely achievable free plasma concentration range [29, 52–54]. Direct extrapolation of total or unbound concentration inhibitory activities in static Vero cell cultures to concentrations in vivo is probably justified only as a rough guide. The antimalarial mode of action of the quinoline antimalarials has been a source of controversy for years [5]. These drugs are weak bases, and they concentrate in the acid food vacuole of the parasite. Chloroquine intercalates DNA, but only at concentrations much higher (1–2 μM) than required to kill malaria parasites (10–20 nM). Chloroquine binds to ferriprotoporphyrin IX, a product of haemoglobin degradation, and thereby chemically inhibits haem dimerization. Inhibition of this essential haem detoxification defence mechanism provides a plausible explanation for the selective antimalarial action of these drugs [56]. Chloroquine also competitively inhibits glutathione mediated haem degradation, another parasite detoxification pathway [57].

## Toxicity

Chloroquine and hydroxychloroquine are generally well tolerated. The most common adverse reactions reported are dyspepsia, nausea, occasionally vomiting, visual disturbances (particularly transient accommodation difficulties), and headache [58–62]. The gastrointestinal adverse effects can often be lessened by taking chloroquine with food. Orthostatic hypotension may be accentuated in febrile patients [63].

The main concern with high doses is cardiovascular toxicity [64]. Parenteral chloroquine causes hypotension if administered too rapidly, or when a large dose (5 mg base/kg or more) is given by intramuscular or subcutaneous injection [16, 20]. In addition, chloroquine and hydroxychloroquine (and the structurally related 4-aminoquinoline amodiaquine and the bisquinoline piperaquine) block several different cation channels [65–68]. In voltage-clamped cat ventricular myocytes, chloroquine blocked both inward and outward membrane currents. The order of potency was as follows: inward rectifying potassium current (I_K1_) → rapid delayed rectifying potassium current (I_Kr_) → sodium current (I_Na_) → L-type calcium current (I_Ca–L_) [66]. This combined effect prolongs the cardiac action potential duration, enhances automaticity, and reduces the maximum diastolic potential [69]. Chloroquine blocked the rapid component of the delayed rectifying potassium current, I_Kr_, but not the slow component, I_Ks_. These different effects explain the prolongation of the electrocardiograph (ECG) QRS and JT intervals. The blockade of I_Na_ and I_Ca–L_ reduce the early afterpotentials that trigger ventricular arrhythmias. Chloroquine and hydroxychloroquine also block the hyperpolarization-activated funny current (I_f_), which plays a major role in the sino-atrial node pacemaker and so may cause bradycardia [68]. Blockade of the I_Kr_ (hERG) channel, which delays ventricular repolarization and thereby prolongs the ECG QT interval, has been the primary focus of concern [70–72]. This is a risk factor for polymorphic ventricular tachycardia (torsade de pointes [TdP]), although there is much debate about the determinants of the risk relationship [73] and the potential ameliorating effects of multichannel blockade [74]. Although chloroquine and hydroxychloroquine do prolong the ECG J to T-peak interval and are potentially ‘torsadogenic’, the extent to which the risk of TdP is increased is unclear. Furthermore, reports of QT prolongation commonly omit measurement of QRS prolongation and thus overestimate JT prolongation. Despite the extensive use of these drugs, there are few case reports of TdP. A Taiwanese patient with systemic lupus erythematosus (SLE), asthma, cirrhosis, and hepatoma developed recurrent TdP during chronic dosing with hydroxychloroquine [70]. In the treatment of COVID-19, TdP developed in an 84-year-old Israeli woman with metastatic breast cancer who was taking bisoprolol, letrozole, and memantine and received chloroquine treatment [75], and also in a 68-year-old US male given hydroxychloroquine and azithromycin [76]. None of these episodes were fatal. The World Health Organization (WHO) pharmacovigilance database (VigiBase) contains reports of 83 episodes of TdP or other forms of ventricular tachycardia that were associated with hydroxychloroquine over a 52-year period, of which seven were fatal. Using data from US poison control and adverse effect reporting databases, the US Food and Drug Administration (FDA) has identified four cases of TdP and 14 cases of ventricular arrhythmia in chloroquine- or hydroxychloroquine-treated COVID-19 infections, although causality is uncertain and the denominator is unknown (as of May 22, 2020, the Strategic National Stockpile had dispensed approximately 2.4 million 7-day treatment courses of hydroxychloroquine to state and local health authorities) [77].

In a recent retrospective self-controlled case series analysis of observational data on 956,374 rheumatoid arthritis patients starting treatment with hydroxychloroquine, there was a lower risk of arrhythmia in the first 30 days of treatment (calibrated hazard ratio [CalHR] 0.89; 95% confidence interval [CI] 0.77–1.04) compared with sulphasalazine recipients (*n* = 310,350) and no effect on mortality (CalHR 0.76; 95% CI 0.44–1.32). In contrast, there was a strong signal for harm when azithromycin was added to hydroxychloroquine, with an increased 30-day cardiovascular mortality (CalHR 2.19; 95% CI 1.22–3.94) and, over the longer term, there was evidence for an increase in cardiovascular mortality [78].

Confusion over toxicity has seriously hampered clinical trials of hydroxychloroquine and chloroquine in COVID-19 infections. This began on May 22, 2020, when a large retrospective observational study reported strong associations between their use and ventricular tachycardia and death in hospitalised COVID-19 patients [79]. It now transpires that the data were almost certainly fabricated, and the paper has been retracted. Nevertheless, despite immediate major concerns over the study's validity (which prompted 201 researchers to write an open letter to the authors and to *The Lancet* [80]), regulatory authorities in the UK (Medicines and Healthcare products Regulatory Agency [MHRA]) immediately advised pausing of all trials involving hydroxychloroquine. The French authorities did the same. Shortly afterwards, WHO suspended recruitment to the hydroxychloroquine arm of the SOLIDARITY trial, which made headline news across the world. In response, the data monitoring committee (DMC) of the largest randomised controlled trial (RCT) in hospitalised COVID-19 patients (RECOVERY: a UK-based trial that had randomised 10,680 patients and enrolled over 1,500 patients in the hydroxychloroquine arm) conducted an emergency review of their unblinded data. This concluded that the trial should continue. Shortly afterwards, the hydroxychloroquine arm of RECOVERY was stopped because of lack of efficacy (not toxicity).

TdP may occur in overdose (see section on chloroquine poisoning), but other arrhythmias usually predominate. There is no evidence for significant risk of TdP in acute treatment with the doses that have been used in malaria or rheumatological conditions [61, 62]. Sudden, unexplained death has not been associated with chloroquine previously, despite administration of billions of prophylaxis and treatment courses and wide variation in dosing [72, 73]. Recent prospective studies providing data from 200,000 patients treated with the related bisquinoline antimalarial compound piperaquine (which has similar hERG channel blocking properties) found no increased risk of TdP after standard treatment [81]. Thus, overall, the concerns that chloroquine or hydroxychloroquine given alone in currently recommended doses are likely to provoke TdP, which have seriously hindered studies in COVID-19, are largely unfounded. In contrast, chloroquine clearly does have beneficial anti-arrhythmic properties, which are underrecognised [82–86]. This is particularly useful in SLE, in which arrhythmias are common and patients on hydroxychloroquine are relatively protected [87]. In laboratory studies, chloroquine and hydroxychloroquine have also been shown to reduce myocardial ischaemia reperfusion injury [88, 89]. Confusion and concern have arisen by extrapolating from the undoubted cumulative long-term risks of myocardial damage with chronic dosing to short-term exposures, overestimating the risk of ventricular arrhythmias resulting from moderate QT prolongation and, in COVID-19 treatments, underestimating the significant contribution of azithromycin to arrhythmia risk. The whole subject has become highly charged and politicised. Unfortunately, overreactions to ‘cardiotoxicity’ case reports and observational data have been allowed to impede conduct of the very randomised controlled trials needed to provide the robust evidence on risks and benefits.

Disease-toxicity interactions may well be relevant. Malaria and malarial fever have independent effects on the QT interval and heart rate [90], although the heart is relatively spared even in severe malaria [91]. There is increasing evidence for myocarditis in COVID-19 [92]. Approximately 5% of hospitalised patients have ventricular arrhythmias. It is unclear whether the mechanism for the cardiotoxic hydroxychloroquine-azithromycin interaction is explained only by TdP or whether there is a febrile illness interaction. It remains to be seen whether patients receiving high-dose chloroquine or hydroxychloroquine for COVID-19 will have more or fewer arrhythmias or other adverse cardiovascular effects than those not receiving 4-aminoquinoline drugs. This is best assessed from RCTs. The evidence reported to date is reassuring. Hypokalaemia is a consistent feature of chloroquine poisoning [93]. Monitoring for cardiovascular adverse events (QRS widening, QT prolongation, arrhythmias) and modifiable risk factors (i.e., plasma concentrations of potassium/calcium/phosphate/magnesium, severely impaired renal function and coadministration of drugs which prolong the QT interval) is advisable in COVID-19 patients receiving high doses of chloroquine or hydroxychloroquine.

Chloroquine and hydroxychloroquine improve glycaemic control in type 2 diabetes and may occasionally cause hypoglycaemia [94–96]. Several factors contribute: stimulation of insulin secretion, reduced insulin degradation, and increased receptor binding. Pruritus is particularly troublesome in dark-skinned patients and may be dose limiting [96]. Itching is described as a widespread prickling sensation mostly affecting the palms, soles, and scalp, which starts within 6 to 24 hours and may last for several days. It can be very distressing. Antihistamine treatment is not usually very effective [97]. Hydroxychloroquine may be associated with less itching. Very rarely, chloroquine may cause an acute and self-limiting neuropsychiatric reaction [58, 61]. Cumulative doses over 100 g (>5 years prophylaxis) are associated with an increased risk of retinopathy [98–103]. Retinal signs include a pale optic disc, arteriolar narrowing, peripheral retinal depigmentation, macular oedema, retinal granularity and oedema, and retinal pigmentary changes consisting of a circle of pigmentation and central pallor, the so-called ‘doughnut’ or ‘bull's eye’ macula. Chloroquine and hydroxychloroquine have also been associated with keratopathy, ciliary body dysfunction, and lens opacities. Reversible corneal opacities can be seen in 30%–70% of rheumatology patients within a few weeks of high-dose treatment. Half are asymptomatic, but some patients may complain of photophobia, visual halos around lights, and blurred vision. Hydroxychloroquine has been considered slightly less toxic to the retina than chloroquine, although with sensitive techniques, retinal damage is evident earlier than appreciated previously [102, 103]. Myopathy is rare at the doses used in antimalarial prophylaxis. Long-term high dose use in rheumatological conditions may cause skeletal or cardiac myopathy. The most commonly reported manifestations (85%) are conduction disturbances, but some patients develop a hypertrophic restrictive cardiomyopathy leading to congestive heart failure [104, 105]. Less common cutaneous side effects include lightening of skin colour, various rashes, photoallergic dermatitis, exacerbation of psoriasis (severe psoriasis is probably a contraindication), bullous pemphigoid, exfoliative dermatitis, pustular rash, skin depigmentation (with long-term use), and hair loss [61]. Contrary to some recent reports, chloroquine and hydroxychloroquine do not cause oxidant haemolysis in glucose-6-phosphate dehydrogenase (G6PD) deficiency [106]. Slight increases in methaemoglobin concentrations follow chloroquine administration in NADH methaemoglobin reductase deficiency [107], but otherwise the 4-aminoquinolines do not cause methaemoglobinaemia. Reports of haemolytic anaemia in G6PD deficient patients hospitalised with COVID-19 infections are best explained by disease-induced haemolysis.

## Drug interactions

Although chloroquine and hydroxychloroquine are metabolised by several of the cytochrome P450 subfamilies (2C8, 3A4/5, 2D6), this is mainly desethylation—and the metabolites are biologically active [12, 14, 43, 44, 93]. Both drugs have some inhibitory activity on these enzymes, but this has not led to clinically significant pharmacokinetic interactions. Renal tubular secretion of chloroquine involves the multidrug and toxin extrusion protein 1 (MATE1), so concomitant administration of MATE1 inhibitors would be expected to reduce renal elimination, but no clinically significant consequences have been reported. Displacement from tissue binding sites may occur, and this probably explains the moderate elevation in primaquine concentrations when coadministered with chloroquine [25]. Chloroquine and hydroxychloroquine both inhibit P-glycoprotein (P-gp) efflux pumps and so may increase cyclosporine and digoxin levels. Cimetidine, but not ranitidine, reduces chloroquine clearance. The main drug-drug interactions causing concern are pharmacodynamic interactions with other hERG channel blocking (QT prolonging) drugs—notably, azithromycin, which has been coadministered commonly with high-dose hydroxychloroquine or chloroquine in COVID-19 treatment and clearly augments QT prolongation [52, 78, 108]. Individually, azithromycin use may carry a greater risk of TdP than chloroquine or hydroxychloroquine. If such drugs are added during or shortly after the 4-aminoquinolines have been given, then plasma potassium concentrations should be over 4 mmol/L, calcium and magnesium plasma concentrations should be in the normal range, and ECGs should be monitored for QT prolongation. Lignocaine or mexiletine may be used to reduce excessively prolonged QT intervals and reduce the TdP risk.

## Chloroquine poisoning

Chloroquine and hydroxychloroquine are dangerous in overdose [47, 93, 109–116]. In self-poisoning, nausea, vomiting, diplopia, hypoacusis, and dysphoria are sometimes followed by tremors, athetoid movements, dysarthria, difficulty swallowing, lethargy and drowsiness, and then seizures, coma, hypotension, hypokalaemia, arrhythmias, and ventricular fibrillation. The lethality of chloroquine in overdose is over six times higher than with other drugs [116]. Outcome is dependent on the dose retained, the blood concentrations that result, and the delay in reaching supportive intensive care. Both drugs are absorbed extensively by activated charcoal, which can be used to limit further absorption. In overdose, a variety of arrhythmias have been observed, including sino-atrial and atrioventricular block, bundle branch block, and different ventricular arrhythmias (including TdP). The ECG commonly shows QRS widening and QT prolongation. Hypokalaemia, resulting from intracellular accumulation, is an important complication, an indicator of prognosis, and a contributor to arrhythmias [93]. It has been suggested that diazepam is a specific antidote [109] for chloroquine poisoning, but more recent studies do not support a specific role for this drug above good haemodynamic and ventilatory support [111, 112]. There is a loose relationship between the self-administered chloroquine dose and the resulting blood concentrations. Mortality is proportional to peak blood concentrations. In Clemessy’s large series from Paris, only one of 106 overdose patients with a peak whole-blood chloroquine + desethychloroquine concentration of less than 25 μM died compared with 13 (21%) deaths in 61 patients with higher concentrations [111, 117]. Hypotension, arrhythmias, coma, and acute respiratory distress syndrome (ARDS) all contributed to death. A fatal outcome was associated with hypotension on admission (systolic BP < 80 mm Hg) and an ECG QRS interval >120 milliseconds. Several patients presented with cardiac arrest, and in some others this occurred after thiopental administration (preceding intubation). Clinical evidence of pulmonary oedema was usually absent on admission. In the patients who developed ARDS, it occurred a mean of 17 hours after admission to the intensive care unit [111]. The plasma potassium concentration on admission correlated inversely with QRS widening and QT prolongation. Good intensive care with prompt management of hypokalaemia were important contributors to survival [113–115]. Patients given large amounts of potassium should be monitored carefully for later rebound hyperkalaemia. There is less information on hydroxychloroquine in overdose, but the complications and the management are similar. Chloroquine and hydroxychloroquine should be stored in secure containers out of reach of children. Chloroquine should not be prescribed to patients with a history of suicide or those who have suicidal ideas.

**Fig 3:**
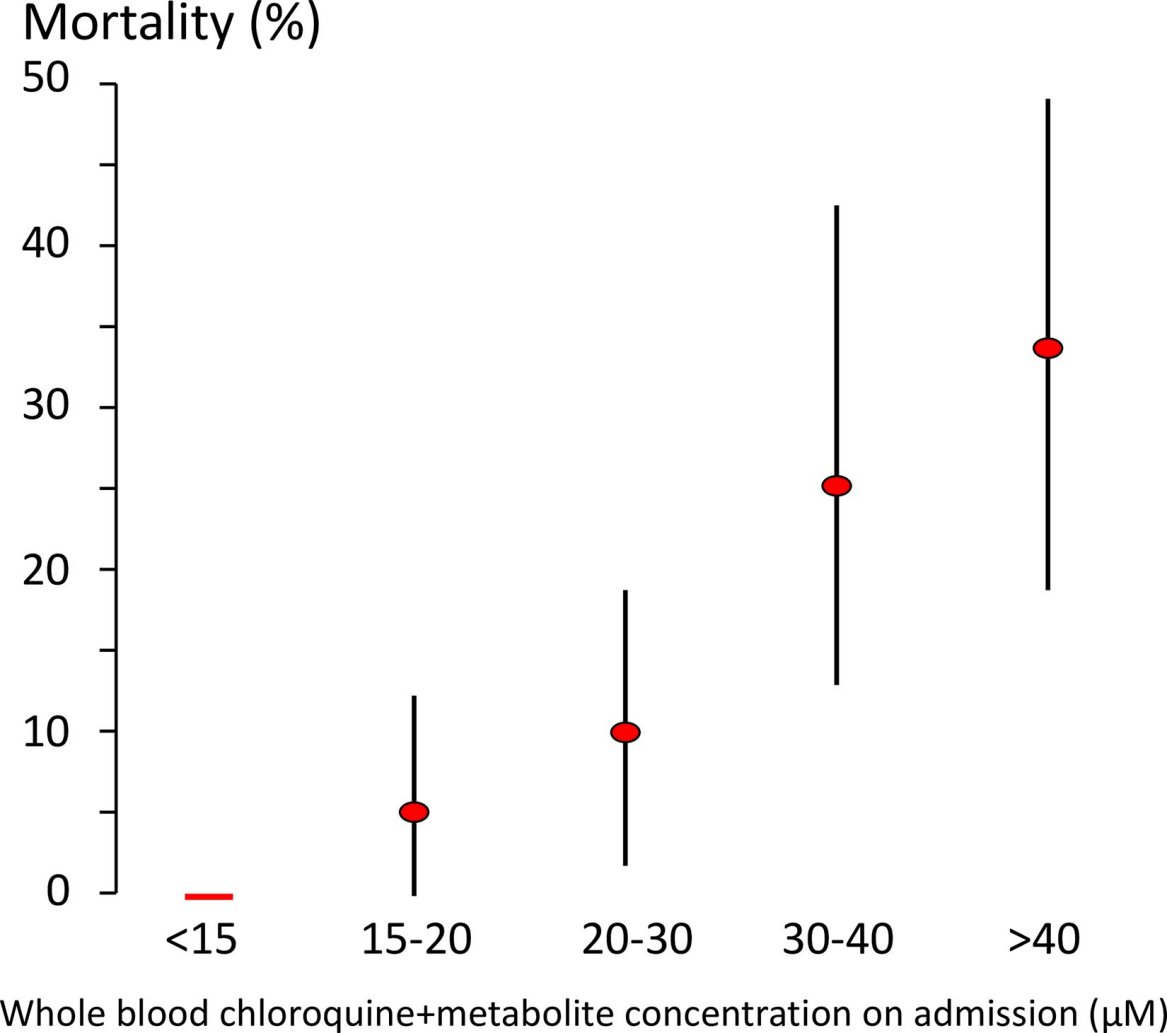
Relationship between whole-blood concentrations of chloroquine + desethyl metabolite (μM) measured by UV-spectrophotometry on admission in self-poisoning, and outcome. The data are from all patients studied prospectively from [93, 109, 111] (*n* = 258). The 95% nonparametric bootstrap confidence intervals are shown by the vertical lines. No deaths were observed for admission concentrations less than 15 μM. Raw data used to produce this image are provided in S1 File.

We pooled individual patient data from prospectively studied large chloroquine self-poisoning cohorts in France (*n* = 258, see S2 Text, data provided in S1 File) [93, 109, 111]. Whole-blood chloroquine concentrations were measured by UV-spectrophotometry, which did not distinguish the parent compound from the desethylated metabolites. Admission whole-blood chloroquine concentrations varied from 1.1 to 81 μM. In patients with an admission whole-blood chloroquine concentration less than 15 μM, no deaths occurred (Fig 3). Mortality rose sharply at higher concentrations, with greater than 5% mortality for concentrations above 20 μM. Assuming that approximately 30% of measured concentrations are attributable to desethychloroquine, this suggests an upper safety bound for peak concentrations of approximately 10 μM in whole blood, or approximately 3 μM in plasma.

## Dosing simulations of chloroquine and hydroxychloroquine

### Treatment simulations

**Fig 4:**
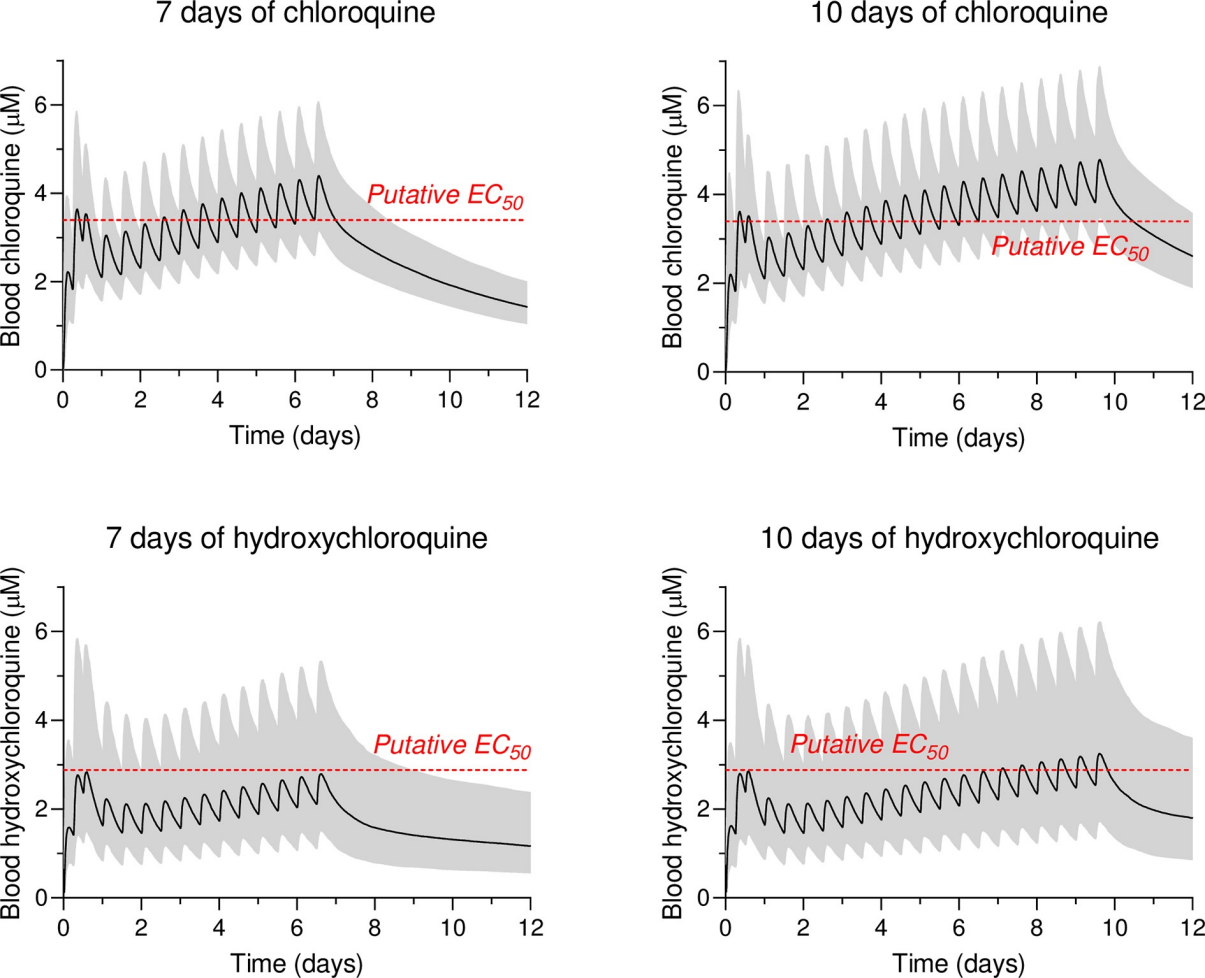
Pharmacokinetic treatment profiles for a typical 65-kg adult. Simulated whole-blood concentration-time profiles of chloroquine (*n* = 1,000), based on [119]. Solid black lines show the population mean concentration-time profiles, and the shaded areas show the 95% prediction intervals. The red dashed line indicates a putative EC_50_ value for SARS-CoV-2, scaled to total blood concentrations (chloroquine: 3.39 μM; hydroxychloroquine: 2.88 μM, using reported in vitro EC_50_ values [29, 54] and a blood-plasma ratio of 3:1 for chloroquine [13, 118] and 4:1 for hydroxychloroquine [38]). These are of uncertain relevance to antiviral activity in-vivo.

We simulated treatment of adult patients with a loading dose of four tablets of hydroxychloroquine sulphate (each tablet contains 200 mg salt, equivalent to 155 mg base) or chloroquine phosphate (each tablet contains 250 mg salt, equivalent to 155 mg base) at time 0 and 6 hours, followed by a maintenance dose of two tablets twice daily (starting 12 hours after the first dose), for a total treatment duration of 7 or 10 days (Fig 4). For a detailed description of the pharmacokinetic modelling, see S2 Text. These are the treatment doses evaluated in the large RECOVERY (10 days’ treatment) and SOLIDARITY (10 days) randomised trials (registered in the ISRCTN registry, numbers 50189673 and 83971151, respectively). The reported in vitro inhibition EC_50_ values for SARS-CoV-2 (1.13 μM for chloroquine [54] and 0.72 μM for hydroxychloroquine [29]), scaled to whole-blood concentrations, are of uncertain relevance to in-vivo activity and are shown only as an approximate indicator. The degree of drug binding in the laboratory cell culture is unknown (most media contain 10% foetal bovine serum with an albumin content of 45–50 g/L). The reported in vitro values [29, 54] were assumed to correspond to total plasma values and scaled to whole blood using a reported blood-plasma ratio of 3:1 for chloroquine [13, 118] and 4:1 for hydroxychloroquine [38], resulting in a putative in vivo blood EC_50_ value of 3.39 μM and 2.88 μM for chloroquine and hydroxychloroquine, respectively. However, only the free fraction of drug can distribute to the site of action (i.e., respiratory epithelium), and the blood-plasma ratios vary widely in the reported literature, so these values are likely to be only a rough approximation of the true in vivo EC_50_ values.

To evaluate exposure and peak whole-blood concentrations in relation to body weights (40–90 kg), we simulated three different dosing strategies shown in S2 Fig:

1. a flat dosing scenario,
2. a weight-based loading dose followed by a flat maintenance dose,
3. a weight-based loading dose and weight-based maintenance dose.

Flat dosing (1) consisted of the 7-day treatment scenario described above (loading dose four tablets twice, maintenance doses two tablets 12 hourly). The weight-adjusted loading doses (2) used three to five tablets to achieve doses as close as possible to 10 mg base/kg loading doses for patients at different body weights, followed by a flat maintenance dose of two tablets 12 hourly to all patients. The third scenario was a weight-adjusted loading dose (as above) and maintenance doses between one and three tablets to achieve 12 hourly maintenance doses as close as possible to 5 mg/kg (S3 Fig and S4 Fig). As expected, weight-based loading and maintenance dosing are preferable, particularly in underweight or overweight patients.

### Prophylactic treatment simulations

**Fig 5:**
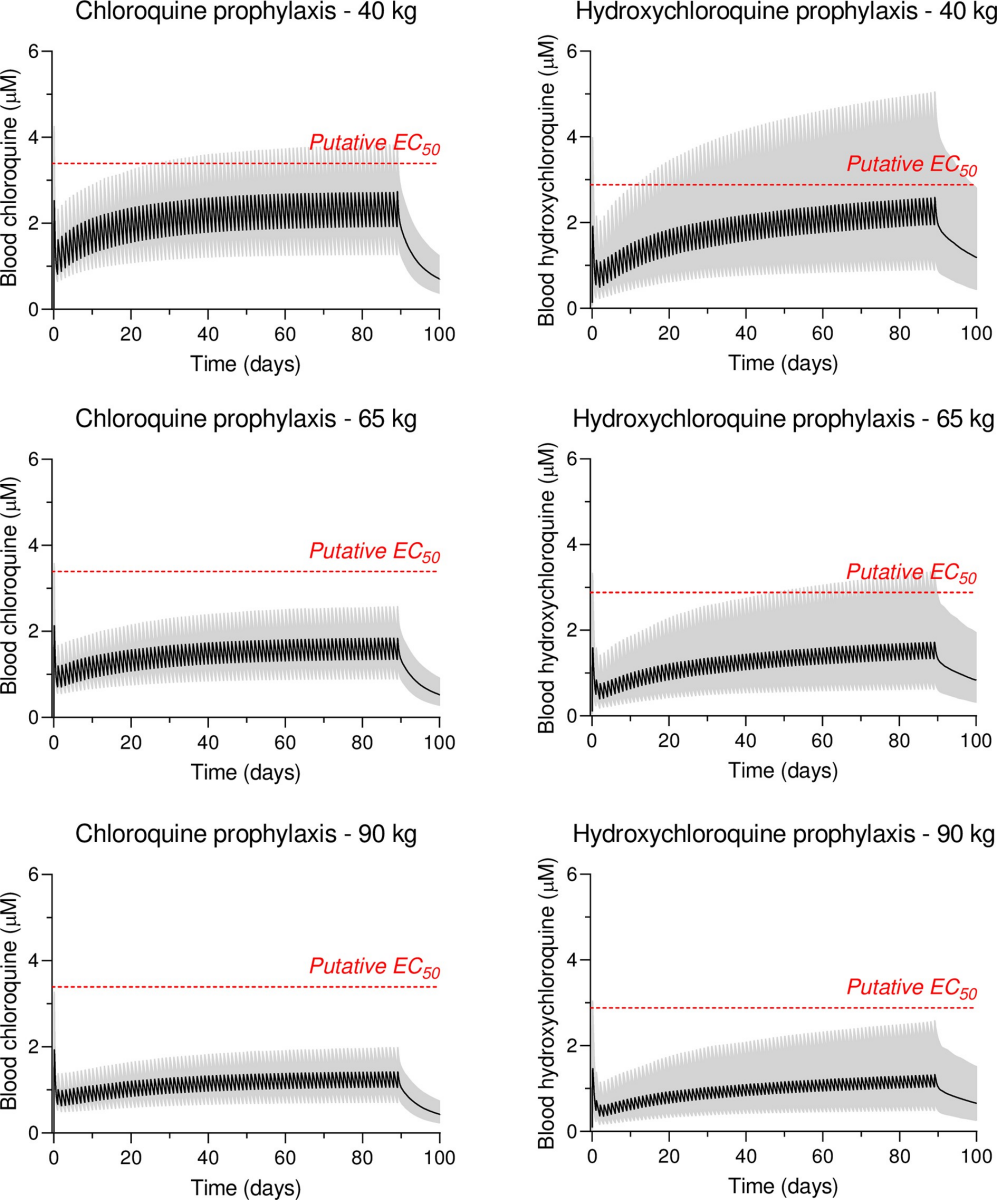
Pharmacokinetic prophylactic profiles of chloroquine and hydroxychloroquine. Simulated whole-blood concentration-time profiles of chloroquine and hydroxychloroquine (*n* = 1,000 per body weight group), based on [119]. Solid black line shows the population mean concentration-time profile and the shaded area shows the 95% prediction interval. Red dashed line indicates a putative EC_50_ value for SARS-CoV-2 as in Fig 4. These EC_50_ estimates derived from in-vitro studies are of uncertain relevance to antiviral activity in-vivo.

Whole-blood exposure and peak concentrations at different body weights (40–90 kg) were simulated using a weight-based loading dose of three to five tablets (S5 Fig) followed by a flat maintenance dose of one tablet daily (Fig 5) for 3 months. These show expected exposures, as in the treatment of rheumatological conditions, which are well below those associated with cardiovascular safety concerns, although it remains to be seen if these levels are effective in the prevention of COVID-19.

### Renal impairment

Renal clearance of chloroquine and hydroxychloroquine has been reported to be between 20% and 55% of total clearance [17, 30]. A ‘worst-case’ scenario was simulated in which renal clearance (50% of total clearance) was reduced by 90%, with no compensatory increase in hepatic clearance. Thus, total body clearance was reduced by 45%. Both acute treatment and prophylactic therapy were simulated, assuming no adjustment in loading dose. An alternative dosing of half the maintenance dose in patients with severe renal impairment is shown (S6 Fig). Whole-blood chloroquine exposure was similar in patients with adequate renal function and renal impairment in short-course treatments of 7 or 10 days. Dose adjustment will not be needed in these patients. Exposures are significantly higher in patients with renal impairment receiving prophylactic treatment. It is uncertain whether this requires dose modification.

## Discussion

Despite the enormous usage of chloroquine in malaria and hydroxychloroquine in rheumatological conditions for over half a century, their clinical pharmacology is not well understood. There are several confusing aspects to their pharmacological assessment. First, dosing is sometimes reported as base equivalent (usually in malaria) and sometimes as salt (rheumatological conditions). Of the 49 different chloroquine or hydroxychloroquine treatment regimens under evaluation for COVID-19 (on clinicaltrials.gov; accessed May 20, 2020), only six explicitly mention the base equivalent. Second, the measurement of these drugs in plasma and blood samples is complicated by extensive binding to platelets, leukocytes, and to a lesser extent, erythrocytes (and in malaria, concentration within malaria parasites). Third, they have complex pharmacokinetic properties characterised by an enormous total apparent volume of distribution and very slow terminal elimination such that blood concentration profiles in acute illness are determined by distribution rather than elimination. Finally, there are still considerable uncertainties about their mode of action.

### The case for evaluating large doses in COVID-19

The moderate inhibitory activity of chloroquine and hydroxychloroquine against the SARS-CoV-2 virus in vitro suggests that if there is any benefit from these medicines, it is likely to require high concentrations of free drug in blood to drive high concentrations in the infected respiratory epithelium. The concentration-effect relationships derived from in vitro studies suggest only partial antiviral effects at best (as shown in Fig 4 and S6 Fig). We assume that the cytosolic concentrations of the drugs in the respiratory epithelium will be in dynamic equilibrium with the free fraction in plasma. We know from the treatment of life-threatening infections that the earlier in the evolving disease process that pathogen multiplication is inhibited, the better is the outcome. This argues for achieving high blood concentrations of chloroquine or hydroxychloroquine as soon as possible in the clinical trials, but this has to be balanced against the potential for serious toxicity. This is exactly the same argumentation used in the treatment of malaria [5], which, in the ‘chloroquine era’, had a mortality of 0.1% for uncomplicated *P*. *falciparum* infections but 15%–20% in severe disease. Serious cardiotoxicity was encountered in a prematurely terminated COVID-19 trial in which the chloroquine malaria treatment loading dose of 10 mg base/kg was given twice daily for a week [120]. In comparison the maintenance doses evaluated in the two largest COVID-19 randomised controlled treatment trials were half this (RECOVERY and SOLIDARITY, ISRCTN registry: 50189673 and 83971151). Provided that there is not a significant drug-disease interaction, these doses evaluated in the two largest RCTs are predicted to be relatively safe (see simulated profiles in Fig 4). Both of these trials have stopped recruitment to the hydroxychloroquine arm because of a lack of evidence for benefit in severe COVID-19. Elsewhere, clinical trials have evaluated smaller doses. For prevention or prophylaxis, where the viral burden is much lower, the daily doses being evaluated are similar to those widely recommended and generally very well tolerated in rheumatological conditions [37, 62, 121–126].

### Making prevention and treatment recommendations before getting the evidence

Many argue that, given the gravity and impact of COVID-19 on individuals, families, organisations, and society, having ‘something is better than nothing’. There is some evidence that chloroquine or hydroxychloroquine ‘work’, so why not use them? Providing medicines for prophylaxis against COVID-19 allays anxiety and gives hope. Giving these drugs to a seriously ill patient gives the impression that something potentially beneficial is being done. And if these drugs do work, then lives would have been saved, and individuals at risk would have been protected. We outline the arguments against recommending this position from the evidence-based medicine perspective that has largely replaced opinion-based medical decision-making. Today, although we now know that hydroxychloroquine is not beneficial in severe disease, we do not know if giving chloroquine or hydroxychloroquine for the prevention or early treatment of COVID-19 is better, or worse, than nothing.

#### Lack of evidence

Publications on COVID-19 are appearing at a rate of hundreds per day, but the only convincing and actionable evidence from large RCTs has come from the RECOVERY trial, which stopped its hydroxychloroquine and lopinavir-ritonavir arms because of lack of efficacy and stopped its dexamethasone arm because of life-saving benefit in patients receiving oxygen or being ventilated. There is also weak evidence that hydroxychloroquine does not work in postexposure prophylaxis, although relatively small but potentially valuable benefits cannot be ruled out by these moderately sized studies [127, 128]. Lack of benefit in hospitalised patients has been extrapolated to lack of any preventive or therapeutic benefit, which is unjustified. Considering the use of chloroquine and hydroxychloroquine in prevention, we are clearly still in a position of substantial uncertainty, awaiting the results of large and definitive studies. These randomised trials are now under substantial threat, as some regulatory agencies have actively stopped ongoing studies, and media opinion, fuelled by a steady stream of observations, cautions, claims, and counterclaims [126], has turned against these highly politicised medicines. Recommending or banning the use of chloroquine or hydroxychloroquine before their safety and efficacy have been well characterised compromises these critical randomised trials, and it violates the generally accepted principle of recommending (or proscribing) interventions only after there is sufficient evidence of their safety and efficacy [129].

#### Risk versus benefits

Chloroquine and hydroxychloroquine are not harmless [71]. Even though we do not know whether they are harmful or beneficial overall in the prevention or treatment of COVID-19, many countries now recommend them for treatment, and seven countries (covering one-fifth of the world's population) have recommended them for prophylaxis in high-risk groups. In contrast, a minority of countries actively promote inclusion of treated patients in clinical trials [130, 131]. A national recommendation based on inadequate evidence is irresponsible, and it gives the public the wrong message. The public, who are understandably desperate for a ‘cure’, will often not read the fine print—or they will believe the deceptive message that ‘something is better than nothing’. This could also lead to widespread self-medication. Fatal self-poisonings have already been reported from unsupervised self-medication of chloroquine and hydroxychloroquine in several countries [131].

#### Diversion

Recommending valuable drugs for unproven indications wastes valuable resources, damages health, and compromises finding effective medicines. People are likely to assume that recommended drugs do work and, in the context of preventive use, will believe they are protected and therefore may not take other necessary precautions or adhere to other public health measures. Taking drugs is easier than complying with public health measures such as physical distancing and wearing protective equipment. In addition, the high demand for these currently unproven drugs has put patients at risk who legitimately need them for treatment for other conditions such as SLE and rheumatoid arthritis. Shortages have already occurred and prices have risen markedly, leaving these vulnerable groups to suffer unnecessarily [132]. It could also encourage unscrupulous manufacturers to make falsified chloroquine and hydroxychloroquine [133].

#### We need good trials

Well-conducted, large, and definitive RCTs are needed [126], but they are not being sufficiently promoted or supported. Premature national recommendations for chloroquine and hydroxychloroquine use or unjustified regulatory statements indicating that these drugs are ineffective in prevention or early treatment have both compromised clinical trials to determine their benefit [134] and made recruitment into these trials more difficult. Several trials have stopped prematurely. In this context, sick patients potentially enrolling in RCTs (and their relatives) will want to know why they are being denied treatments advocated or recommended elsewhere (or conversely given treatments that have been recommended against). Also, potential participants in pre- or postexposure trials may opt for self-medication rather than join the trials. This jeopardises the substantial uncertainty between whether to recommend the drug or not that justifies randomised trials, at least in the eyes of the public.

#### Undermining the drug regulatory system

Recommending chloroquine and hydroxychloroquine for widespread prophylaxis use is not the same as getting approval for unproven drugs for compassionate use. Unjustified recommendations based on opinion or politics rather than evidence undermines public trust in the regulation of the pharmaceutical industry, and it goes against the carefully developed drug approval and regulatory mechanisms established over a generation to protect public safety.

## Supporting information

S1 Text Enantiomer protein binding and kinetics. (DOCX)

S2 Text Pharmacokinetic modelling. (DOCX)

S3 Text Chloroquine self-poisoning cohort data. (DOCX)

S1 Fig **Measured whole-blood concentration data showing the long terminal elimination of chloroquine (top) and hydroxychloroquine (bottom).** Top panel: Measured profiles following 150-, 300-, and 600-mg (base) single oral chloroquine doses reproduced from [14]. Bottom panel: Measured profiles in one individual following 155-mg hydroxychloroquine base intravenous infusion (solid circles) and 310-mg base infusion (hollow squares), reproduced from [30]. The inset in the bottom panel shows the first 100 hours after drug administration. *This is reproduced with permission from the authors [14,30].*
(TIF)

S2 Fig Proposed optimised loading and maintenance treatment doses for COVID-19. (TIF)

S3 Fig Chloroquine pharmacokinetics after different 7-day treatment regimens in COVID-19. Simulated exposures of whole-blood chloroquine, stratified by body weight (*n* = 1,000 per body weight). Solid black line shows the population mean exposure, and the shaded area shows the 95% prediction interval. Black dashed line indicates exposure associated with a standard dosing of 10 mg base/kg loading dose followed by 5 mg base/kg maintenance dose (i.e., exposure in a patient weighing 62 kg). AUC, area under the whole-blood concentration-time curve from time zero to 1 month after the last dose; C_MAX_, maximum concentration.
(TIF)

S4 Fig Hydroxychloroquine pharmacokinetics after different 7-day treatment regimens in COVID-19. Simulated exposures of whole-blood hydroxychloroquine, stratified by body weight (*n* = 1,000 per body weight), based on [119]. Solid black line shows the population mean exposure, and the shaded area shows the 95% prediction interval. Black dashed line indicates exposure associated with a standard dosing of 10 mg/kg loading dose followed by 5 mg base/kg maintenance dose (i.e., exposure in a patient weighing 62 kg). AUC, area under the concentration-time curve from time zero to 1 month after the last dose; C_MAX_, maximum concentration.
(TIF)

S5 Fig Chloroquine and hydroxychloroquine pharmacokinetics in COVID-19 prevention. Simulated whole-blood exposures of chloroquine and hydroxychloroquine, stratified by body weight (*n* = 1,000 per body weight), based on [119]. Solid black line shows the population mean exposure, and the shaded area shows the 95% prediction interval. Black dashed line indicates exposure associated with a standard dosing of 10 mg/kg loading dose followed by 2.5 mg/kg maintenance dose (i.e., exposure in a patient weighing 62 kg). AUC, area under the concentration-time curve from time zero to 1 month after the last dose; C_MAX_, maximum concentration.
(TIF)

S6 Fig Chloroquine and hydroxychloroquine pharmacokinetics in COVID-19 treatment and prevention in patients with renal failure. Simulated whole-blood concentration-time profiles of chloroquine (left column) and hydroxychloroquine (right column) for 7-day treatment regimens (top two panels), 10-day treatment regimens (middle two panels), and 90-day prophylaxis regimens (bottom two panels). The simulations are based on [119]. The solid black lines show the predicted mean concentration-time profiles (*n* = 1,000 simulations) in patients with normal renal clearance after standard maintenance doses (treatment: four tablets as a loading dose on hour 0 and 6 followed on hour 12 by a maintenance dose of two tablets twice daily; prophylaxis: four tablets as a loading dose followed by one tablet daily). The solid red lines show the predicted mean concentration-time profiles in patients with severe renal impairment (10% of normal renal function) after the same loading and maintenance doses. The solid blue lines show the predicted mean concentration-time profiles in patients with severe renal impairment (10% of renal function) after half the standard maintenance doses. The black dashed lines indicate the maximum mean concentrations. The red dashed lines indicate putative EC_50_ values for SARS-CoV-2, scaled to total blood concentrations (chloroquine: 3.39 μM; hydroxychloroquine: 2.88 μM, using reported in vitro EC_50_ values [29, 54] and a blood:plasma ratio of 3:1 for chloroquine [13] and 4:1 for hydroxychloroquine [38]). CL_R_, renal clearance (equivalent to 50% of the total clearance); tab, tablets.
(TIF)

S1 File Concentration outcome data from the self-poisoning studies [93, 109, 111]. (CSV)

S1 Table Comparison of animal toxicity of chloroquine and hydroxychloroquine reported by McChesney and colleagues (1983) [9]. (TIF)

S2 Table Chloroquine and hydroxychloroquine blood-to-plasma ratios. This is reproduced from Table 3 by Mégarbane and colleagues 2010 [47], with permission from the authors.
(TIF)

S3 Table Pharmacokinetic parameters for hydroxychloroquine, used for simulating different dosing scenarios. CL/F is the apparent elimination clearance, V_*c*_/F is the apparent volume of distribution of the central compartment, Q/F is the apparent intercompartmental clearance between the central and peripheral compartments, V_*p*_/F is the apparent volume of distribution of the peripheral compartments, k_*a*_ is the absorption rate constant, T_lag_ is the lag time in the absorption phase, and F is the relative oral bioavailability. Between-patient variability, 30%, was added exponentially in all parameters. Allometric scaling of body weight was added.
(TIF)

S1 Alternative Language Summary Points French translation of the Summary Points by JAW. (DOCX)

## Abbreviations

ARDS: acute respiratory distress syndrome
CalHR: calibrated hazard ratio
CI: confidence interval
DMC: data monitoring committee
ECG: electrocardiograph
FDA: US Food and Drug Administration
G6PD: glucose-6-phosphate dehydrogenase
HPLC: high-performance liquid chromatography
MATE1: multidrug and toxin extrusion protein 1
MHRA: Medicines and Healthcare products Regulatory Agency (UK)
MS/MS: tandem mass spectrometry
P-gp: P-glycoprotein
RCT: randomised controlled trial
SLE: systemic lupus erythematosus
TdP: torsade de pointes
WHO: World Health Organization
