# Correction: COVID-19 prevention and treatment: A critical analysis of chloroquine and hydroxychloroquine clinical pharmacology

**DOI:** 10.1371/journal.pmed.1003445

**Published:** 2020-10-23

**Authors:** The PLOS Medicine Staff

## Notice of republication

This article was republished on October 8, 2020, to replace a file that was incorrectly published as S1 Text. The publisher apologizes for the errors. Please view or download this article again to view the correct version of S1 Text.
