# Supplementary material for: COVID-19 prevention and treatment: A critical analysis of chloroquine and hydroxychloroquine clinical pharmacology
Source: PLoS Med. 2020 Sep 3;17(9):e1003252. doi: 10.1371/journal.pmed.1003252 (PMC7470382; doi:10.1371/journal.pmed.1003252)
Supplement: S1 Text — (DOCX) [file pmed.1003252.s001.docx]

**S1 Text. Enantiomer protein binding and kinetics.**

Chloroquine and hydroxychloroquine are dispensed as racemic mixtures. The enantiomers have different pharmacokinetic and pharmacodynamic properties [1, 2]. Hydroxychloroquine is more hydrophilic than chloroquine. Using HPLC assays the estimated mean terminal elimination half-life was longer for (R)-chloroquine (12.3 days) than for (S)-chloroquine (9.8 days) [3,4]. The estimated mean total body clearance was lower and distribution volume was smaller for the (R)-enantiomer (8.16L/h and 3410L, respectively) than for the (S)-enantiomer (14.2L/h and 720L, respectively). For hydroxychloroquine, in 8 patients on chronic dosing, the blood concentration of (R)-hydroxychloroquine also exceeded that of the (S)-enantiomer, with mean (R)/(S) ratio of 2.2 (range 1.6-2.9). The mean enantiomer blood concentration ratio (R)/(S) for the metabolite desethylhydroxychloroquine was 0.45 (range 0.34-0.58) and 0.56 (range 0.35-0.86) for desethylchloroquine suggesting stereoselective metabolism of hydroxychloroquine. (S)-hydroxychloroquine mean (SD) renal clearance from blood was $41\pm11$ ml/min, approximately twice that of (R)-hydroxychloroquine [5,6]. Protein binding was also different for the chloroquine stereoisomers, with opposite preferential binding to human albumin and the "acute phase protein" alpha 1-acid glycoprotein. Total human plasma protein binding was 67% for (S)-chloroquine and 43% for the (R)-enantiomer [7]. The plasma protein binding estimates for hydroxychloroquine are very similar [2]. The (S)-enantiomer of hydroxychloroquine was 64% bound in plasma, while (R)-hydroxychloroquine was 37% bound. The S enantiomers showed relatively greater binding to albumin and lower binding to alpha 1-acid glycoprotein. Thus, in contrast to their avid tissue binding, chloroquine and hydroxychloroquine are not highly bound to plasma proteins.

**References**

[1] Cardoso CD, Bonato PS. Enantioselective analysis of the metabolites of hydroxychloroquine and application to an in vitro metabolic study. Journal of Pharmaceutical and Biomedical Analysis. 2005;37(4):703–708.

[2] McLachlan A, Cutler D, Tett S. Plasma protein binding of the enantiomers of hydroxychloroquine and metabolites. European Journal of Clinical Pharmacology. 1993;44(5):481–484.

[3] Augustijns P, Verbeke N. Stereoselective pharmacokinetic properties of chloroquine and de-ethyl-chloroquine in humans. Clinical Pharmacokinetics. 1993;24(3):259–269.

[4] Ofori-Adjei D, Ericsson O, Lindstrom B, Sjoqvist F. Protein binding of chloroquine enantiomers and desethylchloroquine. British Journal of Clinical Pharmacology. 1986;22(3):356–358.

[5] Tett SE, McLachlan AJ, Cutler DJ, Day RO. Pharmacokinetics and pharmacodynamics of hydroxychloroquine enantiomers in patients with rheumatoid arthritis receiving multiple doses of racemate. Chirality. 1994;6(4):355–359.

[6] McLachlan AJ, Tett SE, Cutler DJ, Day RO. Disposition and absorption of hydroxychloroquine enantiomers following a single dose of the racemate. Chirality. 1994;6(4):360–364.

[7] Bergqvist Y, Churchill FC. Detection and determination of antimalarial drugs and their metabolites in body fluids. Journal of Chromatography B: Biomedical Sciences and Applications. 1988;434(1):1–20.
