# Supplementary material for: COVID-19 prevention and treatment: A critical analysis of chloroquine and hydroxychloroquine clinical pharmacology
Source: PLoS Med. 2020 Sep 3;17(9):e1003252. doi: 10.1371/journal.pmed.1003252 (PMC7470382; doi:10.1371/journal.pmed.1003252)
Supplement: S2 Text — (DOCX) [file pmed.1003252.s002.docx]

**S2 Text. Pharmacokinetic modelling.**

The chloroquine and hydroxychloroquine population pharmacokinetic models published to date usually do not capture fully the terminal elimination half-life and thus the total apparent volume of distribution of these drugs. Furthermore, some have used whole blood measurements and the others used plasma. Few studies have collected blood samples for longer than 30 days after the last dose, and therefore there are insufficient data to describe adequately the complex multi-compartment kinetic properties of these drugs. The resulting 1- or 2-compartment models underestimate the true terminal elimination half-life. This limitation was highlighted by Frisk-Holmberg and colleagues nearly 40 years ago [1] who reported an increasing terminal half-life when data were quantified for up to 250 days after dose, resulting in a doubling of the estimated terminal elimination half-life (27 vs 53 days) as more data were included in the analysis. This mis-specification results in underestimation of the time to reach steady state, and possibly underestimation of the steady state concentrations. Nevertheless, these simplified (mis-specified) models should predict the concentration-time profiles relatively well for short treatment courses, provided the drugs are not absorbed rapidly enough to outpace distribution from the "central compartment" [2]. This is because the terminal elimination phase has little impact on the acute treatment blood concentration profiles. However, the simplified models are inaccurate when simulating long-term prophylactic regimens because they underestimate the elimination phase and therefore the time to reach steady state. In the pharmacokinetic simulations we therefore used models based on data collected for a long duration in order to characterise both the complex distribution of these drugs and their slow elimination. For chloroquine, a robust population 3-compartment pharmacokinetic model was developed based on "dense" plasma samples collected in healthy volunteers [3]. The final population pharmacokinetic model included body weight as a covariate on clearance and volume parameters, scaled allometrically and centred on 61.9 kg. Between-occasion variability in mean transit time was estimated in the model, but was not included in the simulations. Simulated plasma concentrations were scaled to whole blood concentrations by using a fixed blood-to-plasma ratio of 3 [4]. This correction factor was supported further by comparing simulated population mean values (scaled to whole blood) to measured whole drug concentrations from three clinical studies conducted in healthy volunteers and patients with rheumatological conditions [5-7]. In order to generate model-simulated peak whole blood concentrations after single dose administration, two dose administration and three-months of dosing (i.e. steady-state levels), similar to those observed in the published clinical trials, a blood-to-plasma ratio of 2.85, 2.94, 4.20 was estimated (mean value of 3.33). For hydroxychloroquine, a small but detailed study in healthy volunteers was selected [8], reporting a 3-compartment disposition model resulting in a long terminal half-life of 50 days. Mean pharmacokinetic parameters were derived using the true coefficients and exponents presented in the article. We simulated both short course treatments and repeated dosing to steady-state [9,10], to ensure that model-derived simulated mean concentrations captured the unbiased drug measurements reported in the literature. In the case of a deviation, the relative bioavailability was used to scale the model, resulting in a relative bioavailabity of 60%. Between-patient variability was added exponentially to the hydroxychloroquine model (i.e. 30% between-patient variability in all parameters) in order to capture the approximately 4- to 5-fold variability seen in observed whole blood concentrations. Allometric body weight scaling of clearance and volume parameters was implemented, centred on 63.5 kg, in order to simulate exposures in different weight groups. The final pharmacokinetic model parameters are shown in Table S3. For each scenario 1,000 stochastic simulations were performed and the median and 95% CIs were calculated. The 95% CI was chosen to replicate the approximately 4-fold difference observed between minimum and maximum concentrations in healthy volunteers receiving chloroquine [3]. All models were coded and simulated using the pharmacokinetic software NONMEM v.7.4.3 (Icon Development Solution, Ellicott City, MD). Whole blood simulations were compared to a reported *in vitro* EC value for SARS-CoV-2 of 1.13$\mu$M for chloroquine [11] and 0.72$\mu$M hydroxychloroquine [12]. These indicative values were assumed to correspond to total plasma values and scaled to whole blood using a reported blood-plasma ratio of 3:1 for chloroquine [4] and 4:1 for hydroxychloroquine [13], resulting in a putative *in vivo* blood EC value of 3.39$\mu$M and 2.88$\mu$M for chloroquine and hydroxychloroquine, respectively. The pharmacokinetic model written in NONMEM can be found at http://repository.ddmore.foundation/model/DDMODEL00000323.

**References**

[1] Frisk-Holmberg M, Bergqvist Y, Termond E, Domeij-Nyberg B. The single dose kinetics of chloroquine and its major metabolite desethylchloroquine in healthy subjects. European Journal of Clinical Pharmacology. 1984;26(4):521–530.

[2] White N, Watt G, Bergqvist Y, Njelesani E. Parenteral chloroquine for treating falciparum malaria. Journal of Infectious Diseases. 1987;155(2):192–201.

[3] Pukrittayakamee S, Tarning J, Jittamala P, Charunwatthana P, Lawpoolsri S, Lee SJ, et al. Pharmacokinetic interactions between primaquine and chloroquine. Antimicrobial Agents and Chemotherapy. 2014;58(6):3354–3359.

[4] Rombo L, Ericsson O, Alvan G, Lindström B, Gustafsson LL, Sjöqvist F. Chloroquine and desethylchloroquine in plasma, serum, and whole blood: problems in assay and handling of samples. Therapeutic drug monitoring. 1985;7(2):211–215.

[5] Na Bangchang K, Karbwang J, Thomas C, Thanavibul A, Sukontason K, Ward S, et al. Pharmacokinetics of artemether after oral administration to healthy Thai males and patients with acute, uncomplicated falciparum malaria. British Journal of Clinical Pharmacology. 1994;37(3):249–253.

[6] Mzayek F, Deng H, Mather FJ, Wasilevich EC, Liu H, Hadi CM, et al. Randomized dose-ranging controlled trial of AQ-13, a candidate antimalarial, and chloroquine in healthy volunteers. PLoS Clinical Trials. 2007;2(1):e6.

[7] Augustijns P, Geusens P, Verbeke N. Chloroquine levels in blood during chronic treatment of patients with rheumatoid arthritis. European Journal of Clinical Pharmacology. 1992;42(4):429–433.

[8] Tett S, Cutler D, Day R, Brown K. Bioavailability of hydroxychloroquine tablets in healthy volunteers. British Journal of Clinical Pharmacology. 1989;27(6):771–779.

[9] Al-Rawi H, Meggitt S, Williams F, Wahie S. Steady-state pharmacokinetics of hydroxychloroquine in patients with cutaneous lupus erythematosus. Lupus. 2018;27(5):847–852.

[10] Carmichael SJ, Charles B, Tett SE. Population pharmacokinetics of hydroxychloroquine in patients with rheumatoid arthritis. Therapeutic drug monitoring. 2003;25(6):671–681.

[11] Wang M, Cao R, Zhang L, Yang X, Liu J, Xu M, et al. Remdesivir and chloroquine effectively inhibit the recently emerged novel coronavirus (2019-nCoV) in vitro. Cell Research. 2020;30(3):269–271.

[12] Yao X, Ye F, Zhang M, Cui C, Huang B, Niu P, et al. In Vitro Antiviral Activity and Projection of Optimized Dosing Design of Hydroxychloroquine for the Treatment of Severe Acute Respiratory Syndrome Coronavirus 2 (SARS-CoV-2). Clinical Infectious Diseases. 2020;doi:10.1093/cid/ciaa237.

[13] Morita S, Takahashi T, Yoshida Y, Yokota N. Population pharmacokinetics of hydroxychloroquine in Japanese patients with cutaneous or systemic lupus erythematosus. Therapeutic Drug Monitoring. 2016;38(2):259–267.
