# Supplementary material for: COVID-19 prevention and treatment: A critical analysis of chloroquine and hydroxychloroquine clinical pharmacology
Source: PLoS Med. 2020 Sep 3;17(9):e1003252. doi: 10.1371/journal.pmed.1003252 (PMC7470382; doi:10.1371/journal.pmed.1003252)
Supplement: S3 Text — (DOCX) [file pmed.1003252.s003.docx]

**S3 Text. Chloroquine self-poisoning cohort data**

We conducted a literature review of all published case reports and hospital cohorts of chloroquine self-poisoning. We extracted data from 12 case reports (total of 13 patients) for which chloroquine concentrations were given. We extracted graphical data from the large cohort studied by Riou *et al* (Figure 3 in [1]) in which whole blood chloroquine concentrations and outcome could be determined for 102 patients. Data from [1] were extracted using the web version of WebPlotDigitizer (https://automeris.io/WebPlotDigitizer/). In addition, we obtained the original data from [2] and [3] (the whole blood chloroquine concentrations on admission and hospital outcome, , respectively, but as some patients were included in both studies this gave a total of 247 unique observations). A detailed analysis of these data is reported elsewhere [4]. Data from the case reports exhibited significant bias towards patients with high concentrations who survived. We analysed only case reports in which blood or plasma concentrations were obtained ante-mortem as the post mortem redistribution of chloroquine to the blood is unknown.

Of the patients reported in [1], 91 were included retrospectively and 11 were studied prospectively. The data from the retrospectively studied patients did not exhibit the same relationship between concentration and mortality. For this reason, we only included the eleven prospectively studied patients. Due to the likely selection bias in the case reports, and an unknown source of bias in the retrospectively studied patients in [1], we used all the prospectively studied patients to display the relationship between concentration and mortality (a total of 258 unique patient observations, 111 patients were included in both [2] and [3]). All these patients were studied prospectively and treated in the same intensive care unit in Paris and received similar care. Detailed pharmacometric modelling of the relationship between whole blood chloroquine + metabolite concentrations and outcome in self poisoning is presented separately [4]. Patients included in [3] had multiple whole blood chloroquine measurements. The data are provided in S1 File.

For all patients, whole blood chloroquine concentrations were determined using ultraviolet spectrophotometry at a wavelength of 343 nm. This method does not differentiate between chloroquine and the desethyl metabolite. We assumed that chloroquine represents approximately 70% of the total measured concentration 1-6 hours post ingestion of a single dose [5]. The relationship between whole blood chloroquine concentrations and death estimated from these data will be conservative. This is because chloroquine concentrations were measured on admission, and these will often be slightly lower than the peak concentrations [4]. However, the estimated relationship allows for an objective evaluation of the risk of toxicity in high concentrations.

**References**

[1] Riou B, Barriot P, Rimailho A, Baud FJ. Treatment of severe chloroquine poisoning. New England Journal of Medicine. 1988;318(1):1–6.

[2] Clemessy JL, Borron S, Baud F, Favier C, Hantson P, Vicaut E, et al. Hypokalaemia related to acute chloroquine ingestion. The Lancet. 1995;346(8979):877–880.

[3] Clemessy JL, Taboulet P, Hoffman JR, Hantson P, Barriot P, Bismuth C, et al. Treatment of acute chloroquine poisoning: a 5-year experience. Critical Care Medicine. 1996;24(7):1189–1195.

[4] Watson JA, Tarning J, Hoglund RM, Baud FJ, Megarbane B, Clemessy JL, et al. Concentration-dependent mortality of chloroquine in overdose. Elife 2020; 9 : e58631.

[5] Pukrittayakamee S, Tarning J, Jittamala P, Charunwatthana P, Lawpoolsri S, Lee SJ, et al. Pharmacokinetic interactions between primaquine and chloroquine. Antimicrobial Agents and Chemotherapy. 2014;58(6):3354–3359.
