# Supplementary figures and images for: COVID-19 prevention and treatment: A critical analysis of chloroquine and hydroxychloroquine clinical pharmacology
Source: PLoS Med. 2020 Sep 3;17(9):e1003252. doi: 10.1371/journal.pmed.1003252 (PMC7470382; doi:10.1371/journal.pmed.1003252)

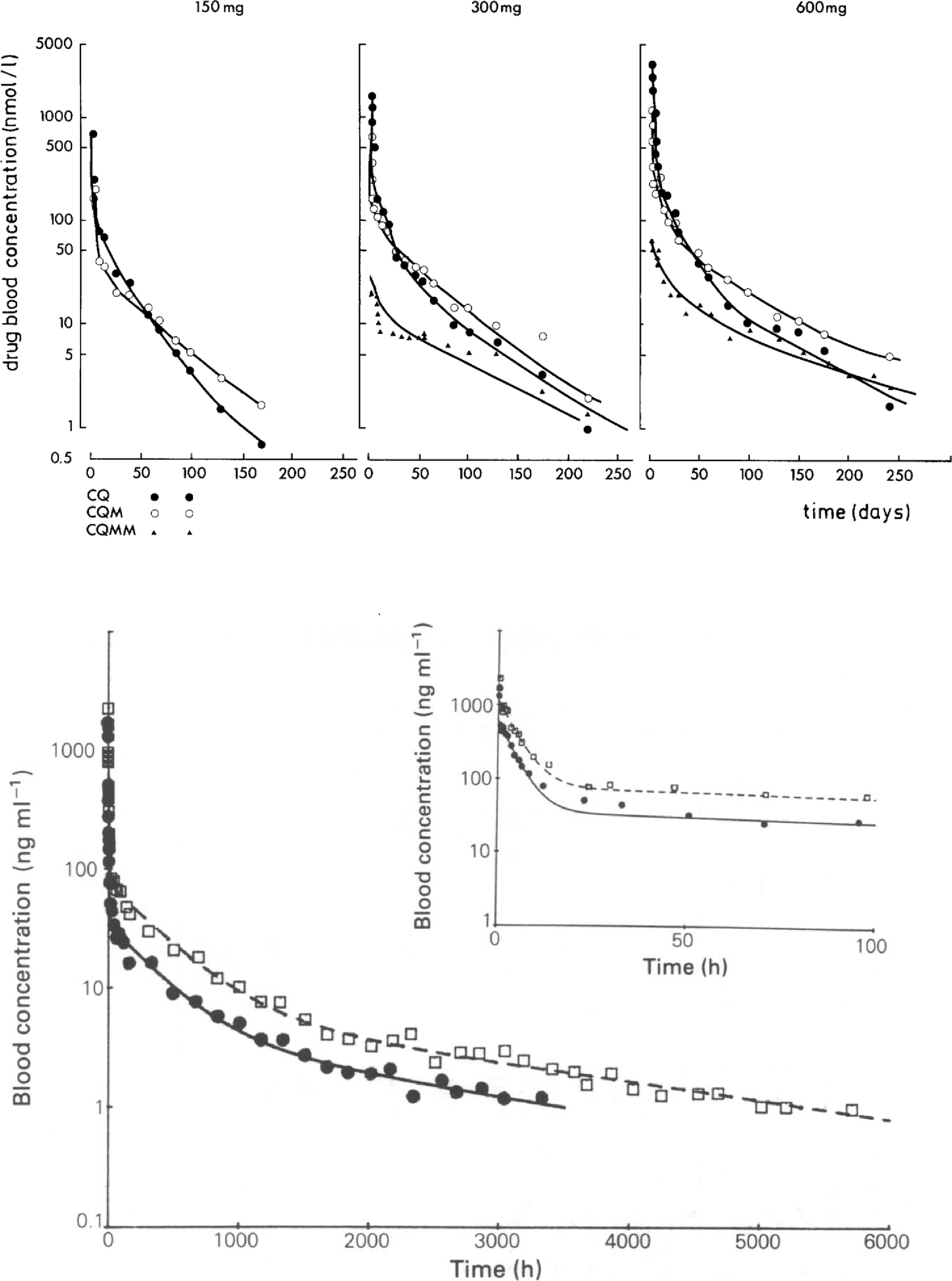

Supplement: S1 Fig — Measured whole-blood concentration data showing the long terminal elimination of chloroquine (top) and hydroxychloroquine (bottom). Top panel: Measured profiles following 150-, 300-, and 600-mg (base) single oral chloroquine doses reproduced from [14]. Bottom panel: Measured profiles in one individual following 155-mg hydroxychloroquine base intravenous infusion (solid circles) and 310-mg base infusion (hollow squares), reproduced from [30]. The inset in the bottom panel shows the first 100 hours after drug administration. This is reproduced with permission from the authors [14,30]. (TIF) [file pmed.1003252.s004.tif]

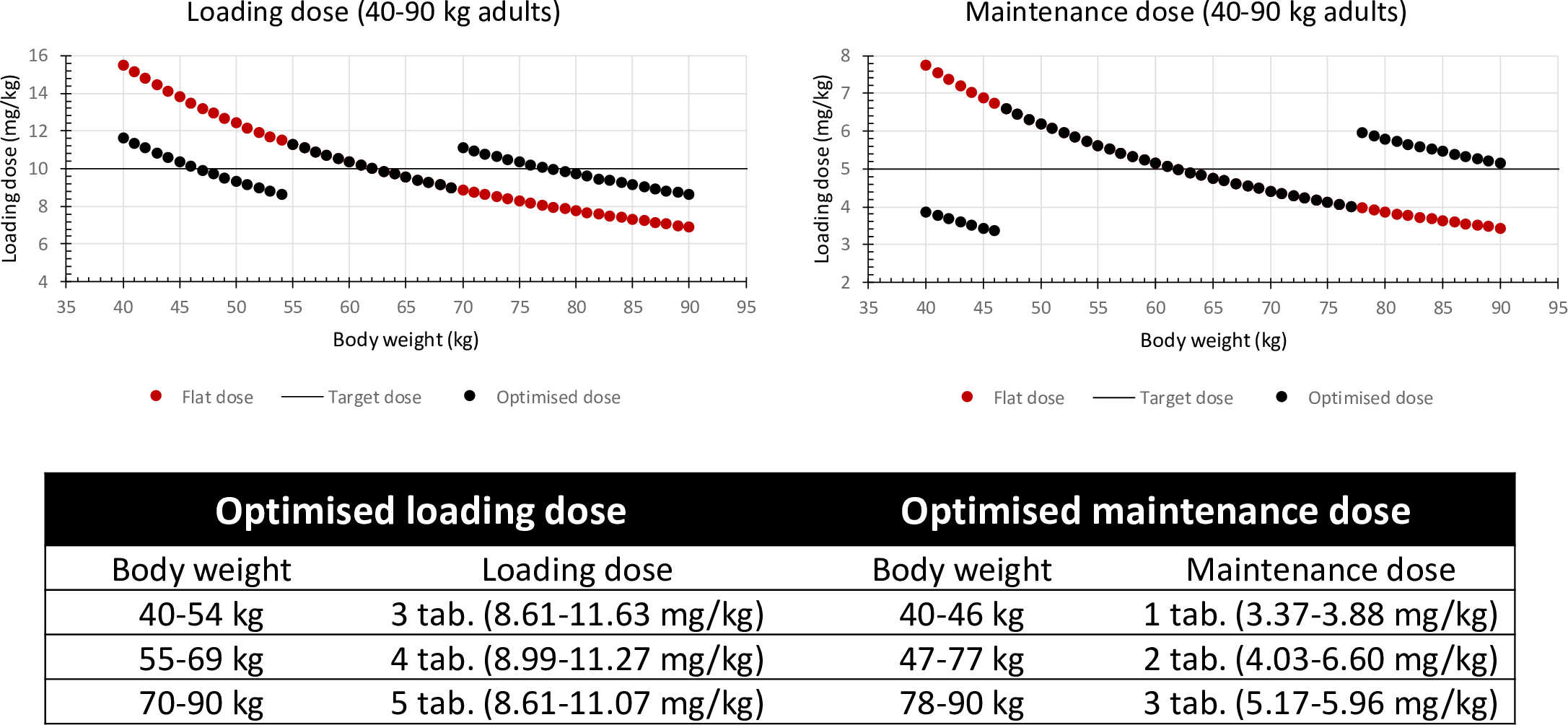

Supplement: S2 Fig — (TIF) [file pmed.1003252.s005.tif]

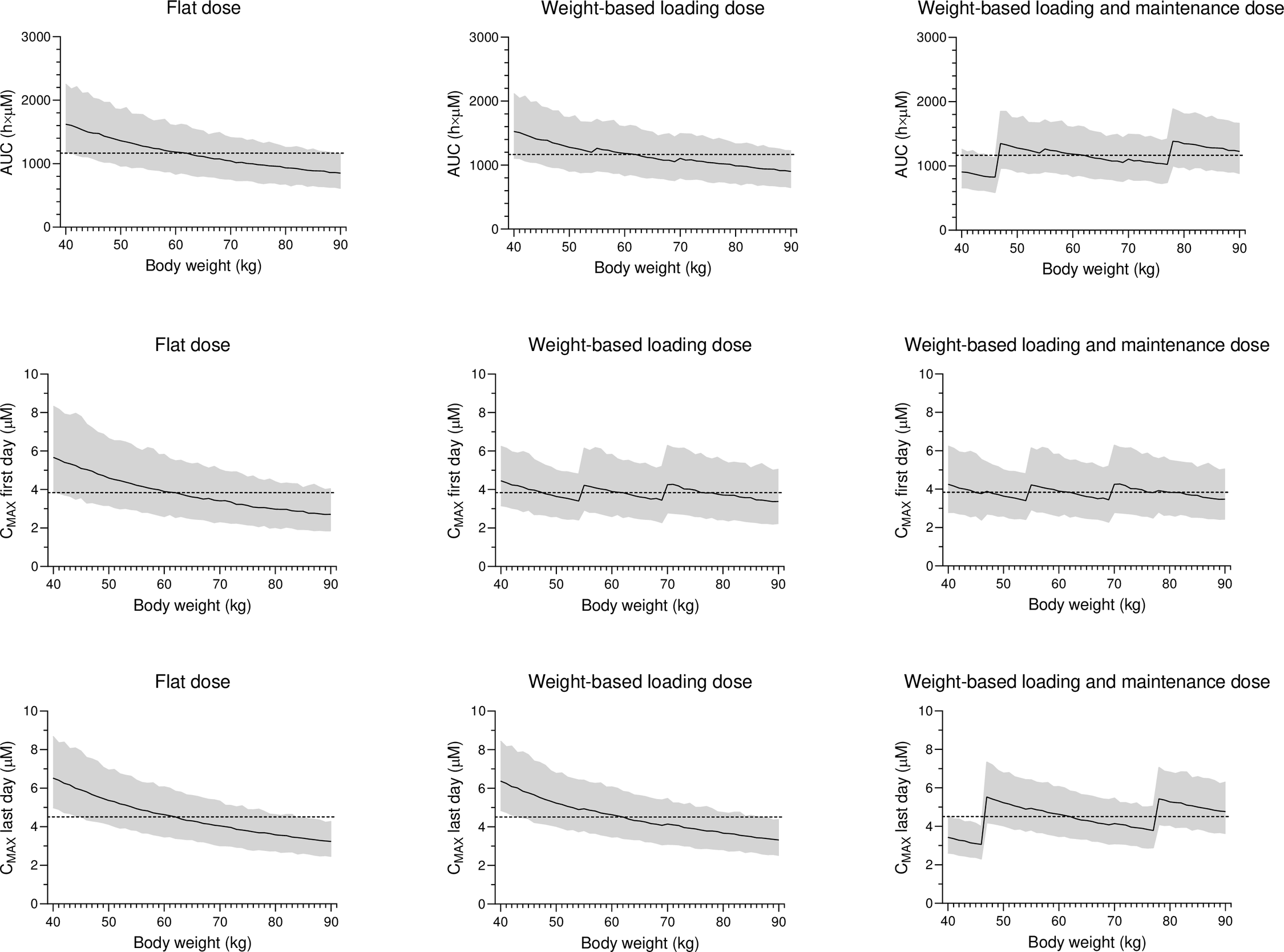

Supplement: S3 Fig — Simulated exposures of whole-blood chloroquine, stratified by body weight (n = 1,000 per body weight). Solid black line shows the population mean exposure, and the shaded area shows the 95% prediction interval. Black dashed line indicates exposure associated with a standard dosing of 10 mg base/kg loading dose followed by 5 mg base/kg maintenance dose (i.e., exposure in a patient weighing 62 kg). AUC, area under the whole-blood concentration-time curve from time zero to 1 month after the last dose; CMAX, maximum concentration. (TIF) [file pmed.1003252.s006.tif]

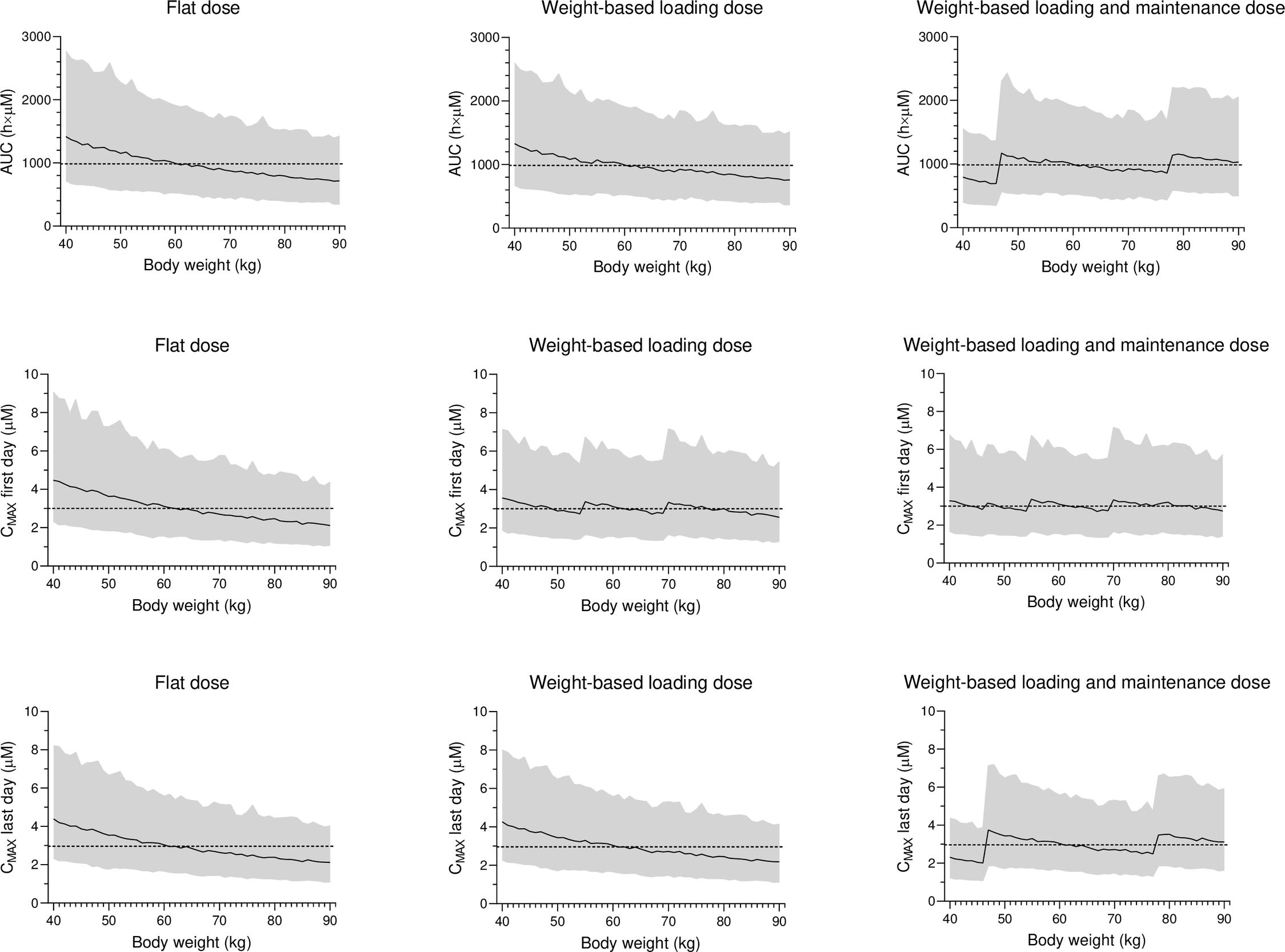

Supplement: S4 Fig — Simulated exposures of whole-blood hydroxychloroquine, stratified by body weight (n = 1,000 per body weight), based on [119]. Solid black line shows the population mean exposure, and the shaded area shows the 95% prediction interval. Black dashed line indicates exposure associated with a standard dosing of 10 mg/kg loading dose followed by 5 mg base/kg maintenance dose (i.e., exposure in a patient weighing 62 kg). AUC, area under the concentration-time curve from time zero to 1 month after the last dose; CMAX, maximum concentration. (TIF) [file pmed.1003252.s007.tif]

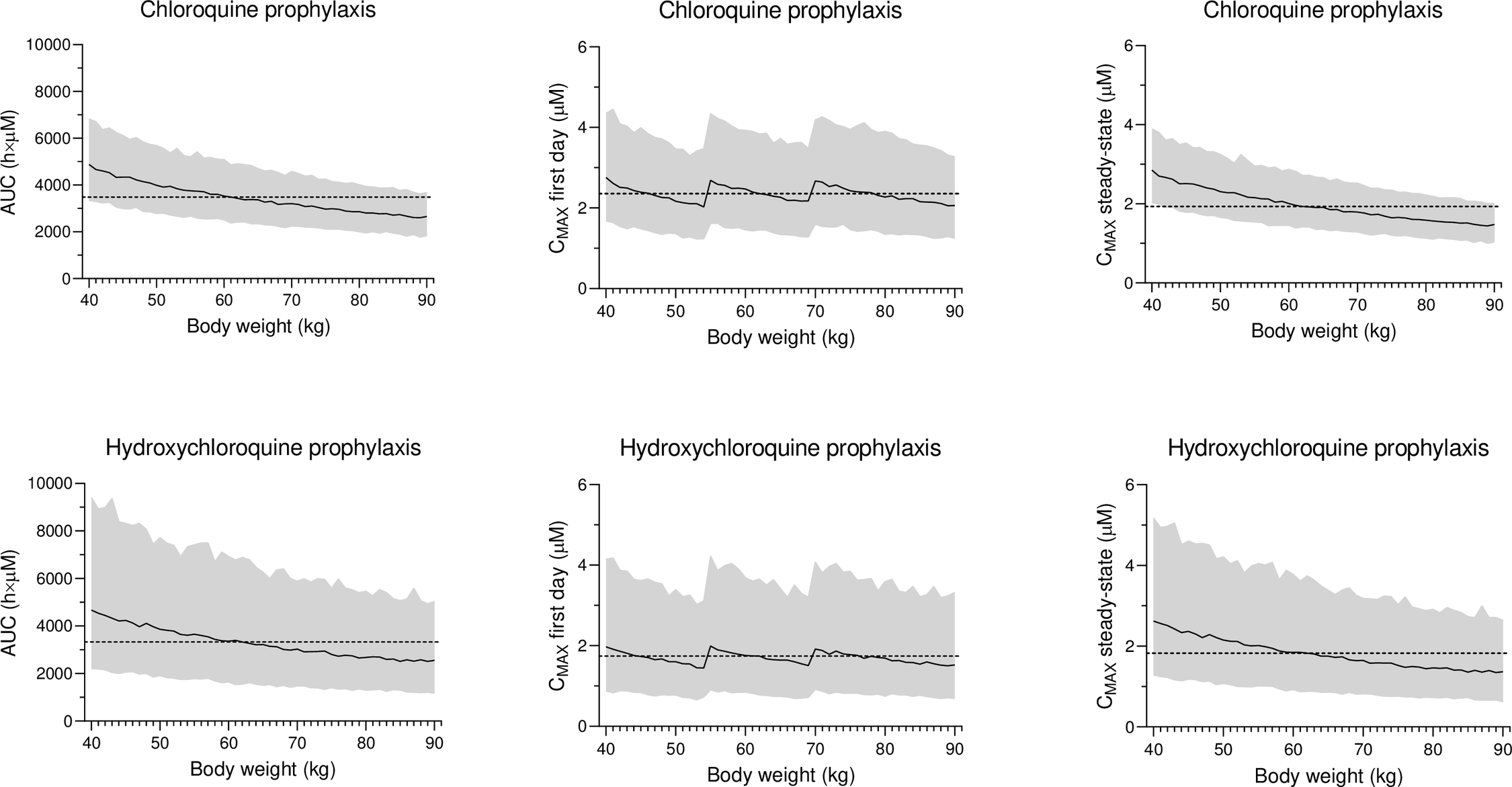

Supplement: S5 Fig — Simulated whole-blood exposures of chloroquine and hydroxychloroquine, stratified by body weight (n = 1,000 per body weight), based on [119]. Solid black line shows the population mean exposure, and the shaded area shows the 95% prediction interval. Black dashed line indicates exposure associated with a standard dosing of 10 mg/kg loading dose followed by 2.5 mg/kg maintenance dose (i.e., exposure in a patient weighing 62 kg). AUC, area under the concentration-time curve from time zero to 1 month after the last dose; CMAX, maximum concentration. (TIF) [file pmed.1003252.s008.tif]

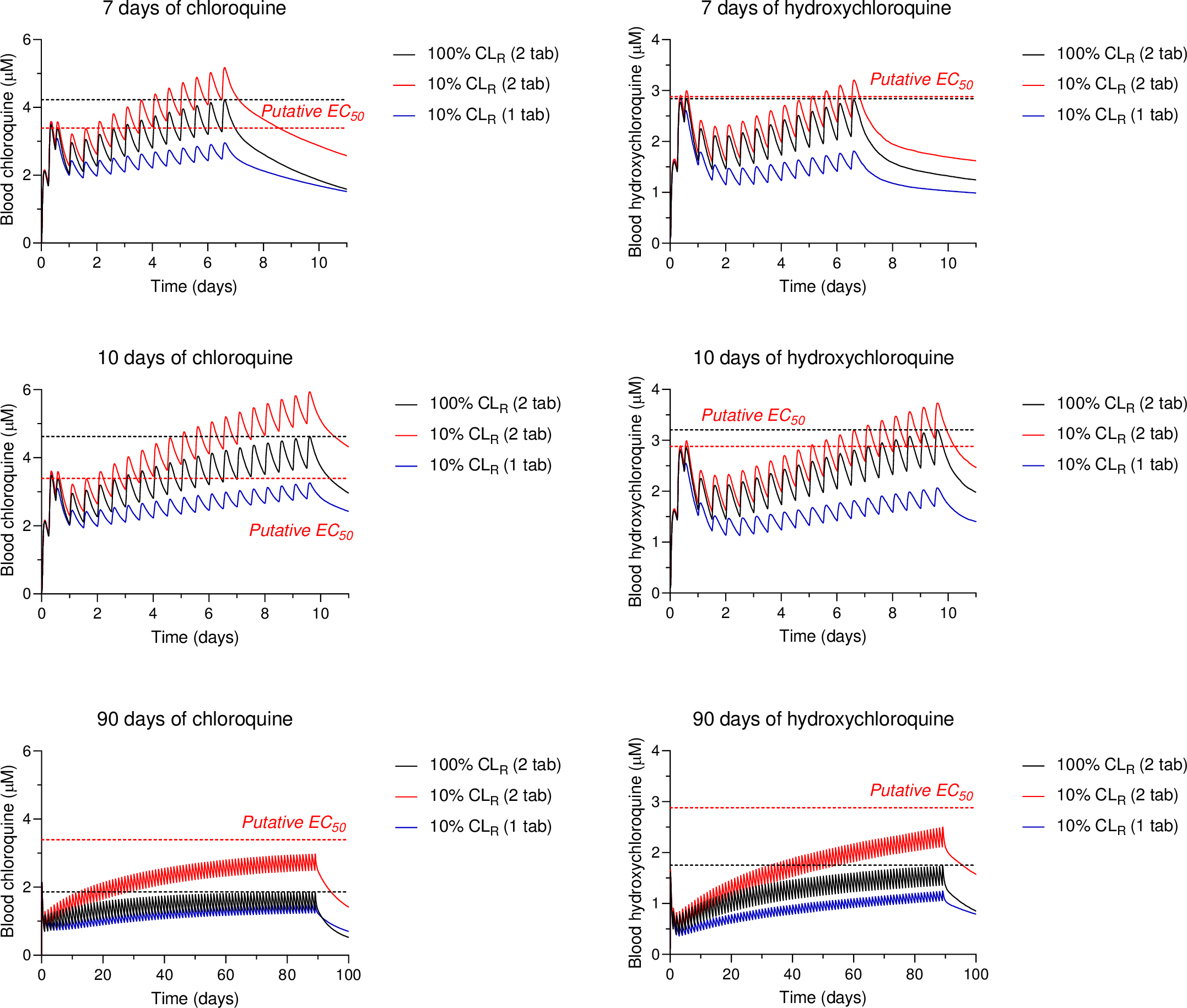

Supplement: S6 Fig — Simulated whole-blood concentration-time profiles of chloroquine (left column) and hydroxychloroquine (right column) for 7-day treatment regimens (top two panels), 10-day treatment regimens (middle two panels), and 90-day prophylaxis regimens (bottom two panels). The simulations are based on [119]. The solid black lines show the predicted mean concentration-time profiles (n = 1,000 simulations) in patients with normal renal clearance after standard maintenance doses (treatment: four tablets as a loading dose on hour 0 and 6 followed on hour 12 by a maintenance dose of two tablets twice daily; prophylaxis: four tablets as a loading dose followed by one tablet daily). The solid red lines show the predicted mean concentration-time profiles in patients with severe renal impairment (10% of normal renal function) after the same loading and maintenance doses. The solid blue lines show the predicted mean concentration-time profiles in patients with severe renal impairment (10% of renal function) after half the standard maintenance doses. The black dashed lines indicate the maximum mean concentrations. The red dashed lines indicate putative EC50 values for SARS-CoV-2, scaled to total blood concentrations (chloroquine: 3.39 μM; hydroxychloroquine: 2.88 μM, using reported in vitro EC50 values [29, 54] and a blood:plasma ratio of 3:1 for chloroquine [13] and 4:1 for hydroxychloroquine [38]). CLR, renal clearance (equivalent to 50% of the total clearance); tab, tablets. (TIF) [file pmed.1003252.s009.tif]

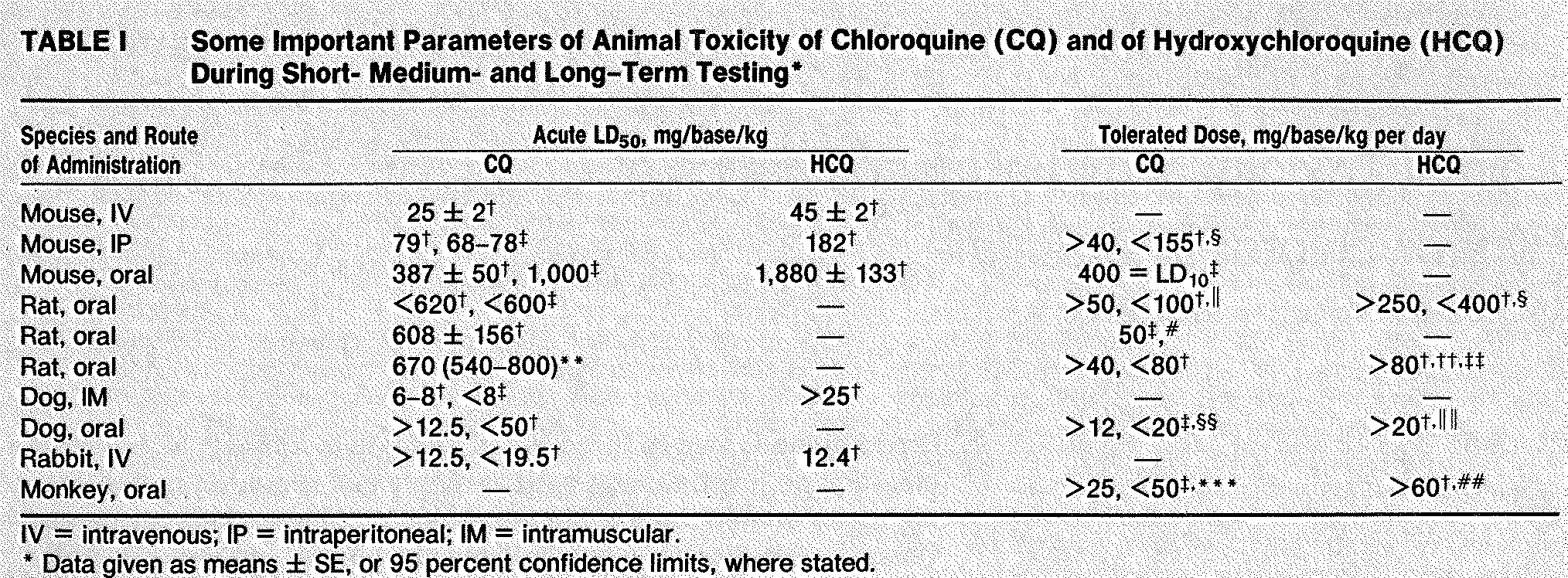

Supplement: S1 Table — (TIF) [file pmed.1003252.s011.tif]

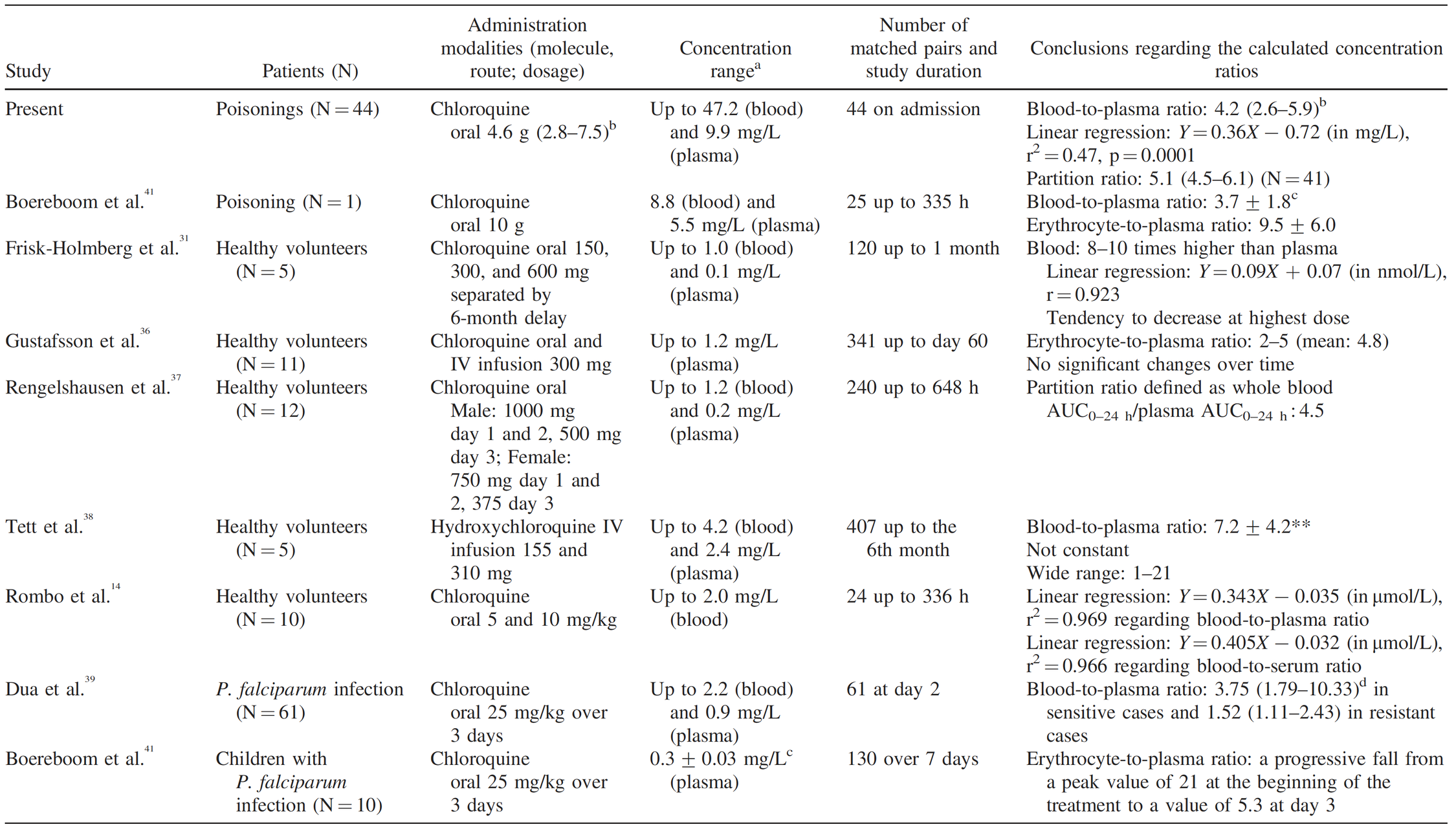

Supplement: S2 Table — This is reproduced from Table 3 by Mégarbane and colleagues 2010 [47], with permission from the authors. (TIF) [file pmed.1003252.s012.tif]

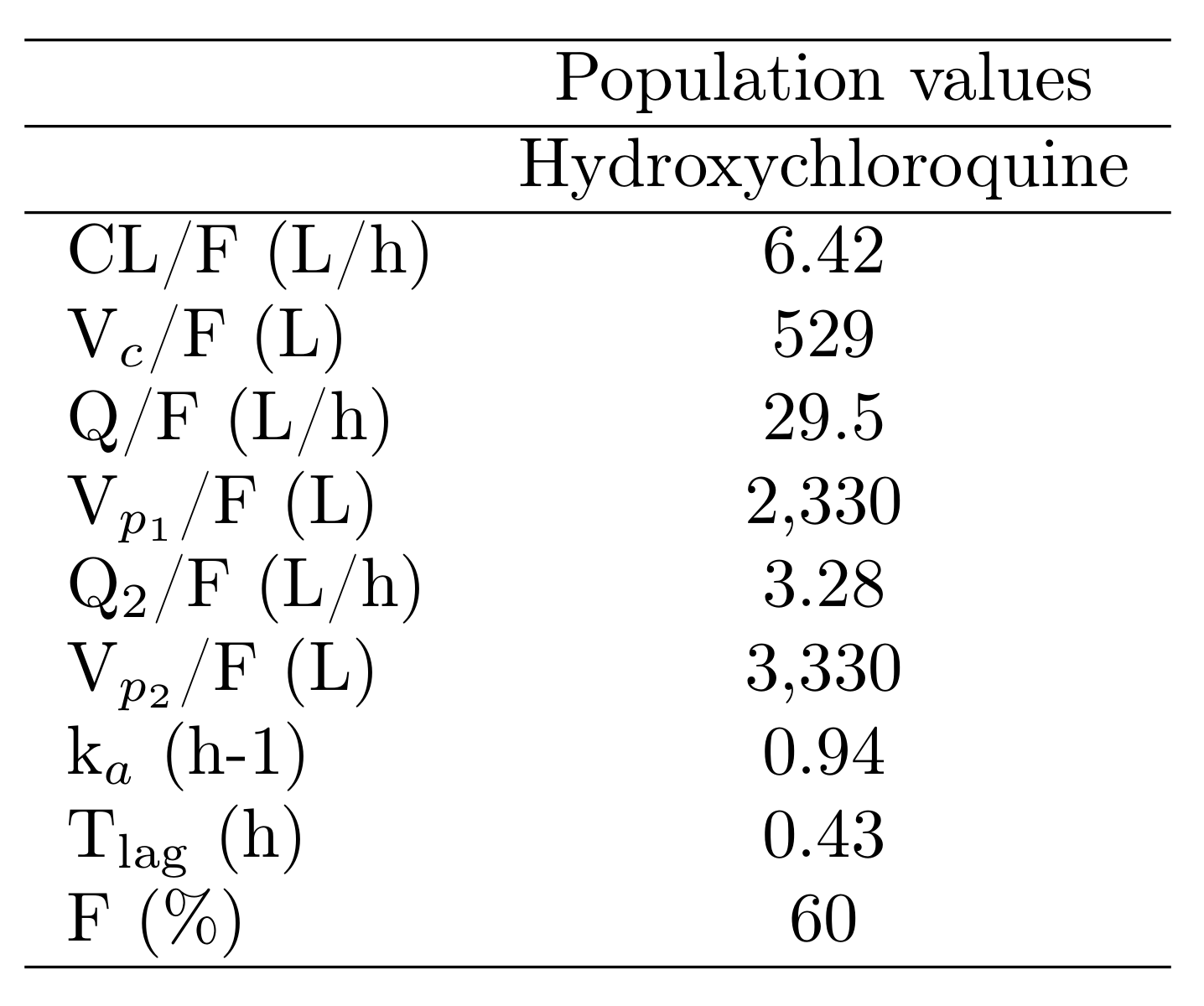

Supplement: S3 Table — CL/F is the apparent elimination clearance, Vc/F is the apparent volume of distribution of the central compartment, Q/F is the apparent intercompartmental clearance between the central and peripheral compartments, Vp/F is the apparent volume of distribution of the peripheral compartments, ka is the absorption rate constant, Tlag is the lag time in the absorption phase, and F is the relative oral bioavailability. Between-patient variability, 30%, was added exponentially in all parameters. Allometric scaling of body weight was added. (TIF) [file pmed.1003252.s013.tif]
