# Supplementary material for: COVID-19 prevention and treatment: A critical analysis of chloroquine and hydroxychloroquine clinical pharmacology
Source: PLoS Med. 2020 Sep 3;17(9):e1003252. doi: 10.1371/journal.pmed.1003252 (PMC7470382; doi:10.1371/journal.pmed.1003252)
Supplement: S1 Alternative Language Summary Points — (DOCX) [file pmed.1003252.s014.docx]

- La chloroquine et l’hydroxychloroquine ont été utilisées pendant plus de 60 ans pour le traitement du paludisme, des abcès amibiens du foie, et de plusieurs maladies rhumatismales, mais leur pharmacologie clinique est mal caractérisée. COVID-19 est une nouvelle indication thérapeutique même si ces médicaments n’ont qu’une activité faible \textit{in-vitro} contre le virus SARS-CoV-2, et il n’y a pas encore de données convaincantes d’efficacité clinique.
- La chloroquine et l’hydroxychloroquine ont des propriétés pharmacocinétiques inhabituelles avec des volumes de distribution très importants (chloroquine > hydroxychloroquine) et une élimination très lente (demi-vies de phase terminale > 1 mois).
- Les concentrations plasmatiques de la chloroquine et l’hydroxychloroquine libres (non liées aux protéines) qui causent des effets indésirables sérieux (hypotension, troubles de la conduction cardiaque, anomalies de la repolarisation ventriculaire, et toxicité neurologique) sont déterminées principalement par les processus de distribution dans le corps.
- L’hydroxychloroquine était légèrement moins toxique que la chloroquine dans les essais précliniques et est considérée comme étant mieux tolérée sur le long terme. Ces deux médicaments sont dangereux en surdosage, et l’administration par voie parentérale requière la plus grande prudence.
- Ces médicaments se présentent sous forme de sels médicamenteux différents, chacun avec une masse en base différente. Ceci peut provoquer une confusion et a parfois entrainé des erreurs de dosages. Les sels médicamenteux varient selon les pays et donc le traitement pour le paludisme est généralement recommandé en termes de base. Les comprimes des deux formulations les plus courantes, chloroquine diphosphate 250 mg sel et hydroxychloroquine sulphate 200 mg sel, contiennent tous deux 155 mg de base.
- Les effets proarythmiques et antiarhythmiques de la chloroquine et de l’hydroxychloroquine ont été peu caractérisés mais la majorité des données concernant les schémas thérapeutiques en cours d’essai sont rassurants. Les risques d’arythmie cardiaque ont été inférés à partir de l’allongement de l’intervalle QT plutôt qu’observés directement.
- Nous avons utilisé des données disponibles chez des volontaires sains, dans le traitement du paludisme, ou des maladies rhumatismales et de la toxicocinétique de la chloroquine après empoisonnement, pour prédire l’exposition systémique et les marges de sécurité des schémas thérapeutiques à haute dose utilisés pour la prévention et le traitement du COVID-19.
- Ces schémas thérapeutiques ont une marge de sécurité raisonnable. Des doses plus faibles pourraient ne pas être efficaces contre cette infection parfois mortelle. Tout schéma thérapeutique devrait éviter d’atteindre des concentrations sanguines supérieures à 10uM (3.2ug/mL), ce qui correspond approximativement à des concentrations plasmatiques >3uM (1ug/mL).

Des essais randomisés de grande taille et bien exécutés avec une surveillance appropriée sont requis pour déterminer si la chloroquine et l’hydroxychloroquine sont efficaces pour la prévention ou le traitement du COVID-19, avec une bonne tolérance. Des recommandations en dehors d’essais cliniques ne sont pas justifiées pour le moment.
